# Use of Telenursing in the conservative treatment of patients with chronic renal insufficiency: scoping review ^*^

**DOI:** 10.1590/1518-8345.7013.4359

**Published:** 2024-10-25

**Authors:** Aline de Oliveira Biancamano, Alessandra Conceição Leite Funchal Camacho, Elaine Antunes Cortez, Harlon França de Menezes, Yasmin Saba de Almeida, Cristiele Costa da Matta Rocha

**Affiliations:** ^1^ Universidade Federal Fluminense, Escola de Enfermagem Aurora de Afonso Costa, Niterói, RJ, Brazil.; ^2^ Ministério da Saúde, Hospital Federal dos Servidores do Estado, Serviço de Nefrologia, Rio de Janeiro, RJ, Brazil.; ^3^ Universidade Federal Fluminense, Escola de Enfermagem Aurora de Afonso Costa, Departamento de Fundamentos de Enfermagem e Administração, Niterói, RJ, Brazil.; ^4^ Universidade Federal Fluminense, Escola de Enfermagem Aurora de Afonso Costa, Departamento de Enfermagem Materno-Infantil e Psiquiátrica, Niterói, RJ, Brazil.

**Keywords:** Telenursing, Treatment Adherence and Compliance, Self-care, Renal Insufficiency Chronic, Conservative Treatment, Biomedical Technology

## Abstract

**Objective:**

to map the evidence of the use of telenursing in adherence to treatment and promotion of self-care in patients with chronic renal insufficiency undergoing conservative treatment.

**Method:**

scoping review study, with selection of documents indexed in eight databases and two catalogs of theses and dissertations. Additionally, the reference lists of selected studies were consulted. Selection and analysis of studies were carried out using Rayyan software through double, independent and blind investigation.

**Results:**

56 eligible publications were found and ten were selected to compose the study. The following stand out among telenursing interventions: cell phone applications, websites, digital platform, telephone support and teleconferencing.

**Conclusion:**

it was evident that the use of Telehealth by Nursing is relevant to the health sector, but still little explored in national and international literature, representing a gap in knowledge to be filled in future research. Studies have shown that its implementation helps and supports healthcare professionals, providing inferences and guidance for quick, safe and effective assistance, even remotely. Telenursing presents itself as a strategy capable of promoting adherence to treatment and self-care in patients with chronic renal insufficiency undergoing conservative treatment.

## Highlights

**(1)** Develops health innovation strategies in remote treatment.

**(2)** Provides support to professionals with preventive measures, minimizing costs.

**(3)** Encourages the practice of self-management of care.

**(4)** Provides inferences and guidance for quick and safe assistance.

**(5)** Contributes to the advancement of public policies in the area of noncommunicable diseases.

## Introduction

 Chronic renal insufficiency is defined by the presence of changes in the structure or functions of the kidneys, with or without changes in glomerular filtration, for a period longer than three months ^(^ ^1^ ^)^ . It is the glomerular filtration rate (GFR) that has the greatest correlation with clinical outcomes, despite creatinine being the best marker of kidney function ^(^ ^2^ ^)^ . The GFR level varies depending on age, gender and muscle mass. With advancing age, it is possible to observe a decrease in GFR, and this reduction may manifest itself long before the onset of symptoms and be associated with the severity of chronic kidney disease (CKD) ^(^ ^3^ ^)^ . 

 CKD is progressive and irreversible to date, but it can be initially treated through conservative therapeutic measures, which consist of clinical measures used to delay the worsening of kidney function, reduce symptoms and prevent complications ^(^ ^4^ ^)^ . Starting conservative treatment early increases the chances of preserving kidney function for a longer period of time ^(^ ^5^ ^)^ . 

 Conservative treatment addresses the management of risk factors to prevent the progression of kidney disease in its initial stages, which range from 1 to 3, when the GFR is between ≥90 and 30 mL/min/1.73 m ^2^ . It also encompasses the pre-dialysis phase in stages 4 and 5, non-dialysis, when GFR varies from 29 to <15 mL/min/1.73 m ^2^ , and includes renal replacement therapy when dialysis stage 5 is reached, in which GFR is already below 15 mL/min/1.73 m ^2^^(^ ^2^ ^)^ . 

 The patient’s control of CKD is directly related to changes in lifestyle habits, and these are effectively achieved when included in the day-to-day care activities that the person carries out to improve their own health (self-care) ^(^ ^6^ ^)^ . 

 Because the early stages of CKD are typically asymptomatic and easily ignored, awareness of the disease is low, and patients often delay and neglect treatment. Therefore, health professionals who care for users with chronic conditions need new models that help them develop advanced communication skills ^(^ ^3^ ^)^ . 

 With the advent of the pandemic and the need to maintain isolation and social distancing, the Federal Nursing Council of Brazil (Cofen), in 2020 ^(^ ^7^ ^)^ , decided to standardize the practice of nursing teleconsultation in Brazil and, later, in 2022, created Resolution No. 696/2022 ^(^ ^8^ ^)^ , standardizing telenursing with the role of nurses in digital health. Telenursing integrates telehealth and is characterized by the use of technological resources to carry out nursing practice remotely, in the dimensions: assistance, educational or research ^(^ ^9^ ^)^ . 

 From this perspective, an integrative review ^(^ ^10^ ^)^ evaluated the use of telenursing in the care of chronic patients and showed that this technology provided benefits to patients, professionals and the health system. Regarding patients, there was a feeling of empowerment, improved disease management, decreased anxiety, improved quality of life and increased medication adherence. As for professionals, there was an increase in communication and support, better management of symptoms, enabling early diagnosis and intervention, and savings in working time. Finally, for the health system, a reduction in the rate of hospital admissions and outpatient consultations was detected. 

Understanding the potential of telenursing for the care of patients with CKD and the urgency of starting conservative treatment, studies that seek to portray its use become essential for public health. However, the search for a deeper understanding of the topic is still challenging, since there is a scarcity of materials due to the novelty of the practice, and the little literature available is dispersed or difficult to access. Thus, this scoping review is based on the imminence of bringing visibility and understanding to the central concept of the study, supported by the knowledge gap identified through previous searches in Prospero, Cochrane Database of Systematic Reviews and JBI Evidence Synthesis, the results of which demonstrate that there are no revisions, completed or in progress, with this focus.

Therefore, this scoping review is justified, which aims to map the evidence of the use of telenursing in adherence to treatment and promotion of self-care in patients with chronic renal insufficiency undergoing conservative treatment. This study may contribute to the dissemination of a new clinical-practical approach to health promotion in conservative treatment in patients with chronic renal insufficiency.

## Method

### Study design

 This is a scoping review study, according to the review method proposed by the JBI collaboration ^(^ ^11^ ^)^ . This method allows to provide a comprehensive and unbiased synthesis of relevant studies within the confines of a single document, using rigorous and transparent methods ^(^ ^12^ ^)^ . To report the review, the Preferred Reporting Items for Systematic reviews and Meta-Analyses extension for Scoping Reviews (Prisma-ScR) checklist was used ^(^ ^13^ ^)^ . 

Therefore, this investigation’s main objective is to map the evidence on the use of telenursing, guided by the following question: How does the use of telenursing influence adherence to treatment and promotion of self-care in patients with chronic renal insufficiency undergoing conservative treatment? To construct the research question, the mnemonic strategy population, concept and context (PCC) was used. The following were defined: population - people with chronic renal insufficiency undergoing conservative treatment; concept - telenursing; and context - adherence to treatment and promotion of self-care.

### Protocol and registration

 Initially, a research protocol was carried out with the objective of guaranteeing the methodological rigor of the review, and its components comprised the following phases: definition of the objective and research question; definition of eligibility criteria (inclusion and exclusion); planning the search strategy and selection of studies in relation to the specific descriptors of each database; identification of studies and selection of studies; data extraction; data mapping; and summarization of results. The final protocol was registered on the Open Science Framework (OSF) platform ^(^ ^14^ ^)^ . 

### Eligibility criteria

According to the acronym PCC, the following eligibility criteria were established: inclusion - primary cross-sectional, observational studies, clinical trials, quasi-experimental and randomized studies, carried out with adult patients (18 years or older), published in any language, without period delimitation; exclusion - letters to the editor and editorials; studies that only addressed chronic renal insufficiency in conservative treatment, but did not address telenursing; studies that addressed dialysis therapy without conservative treatment as one of the methods or exclusive method; studies that did not have at least one professional nurse working or developing the technology associated with telehealth; and, furthermore, that the approach was not a telehealth device.

It is worth noting that, although secondary studies were initially considered for inclusion in the review, no publication with this design answered the research question.

### Sources of information and research

 The search was carried out on January 6, 2023 and updated on April 23, 2024. First, the following electronic databases were consulted: *Base de Dados em Enfermagem* (BdEnf), *Índice Bibliográfico Español en Ciencias de la Salud* (Ibecs) and Latin American and Caribbean Health Sciences Literature (LILACS), through the Virtual Health Library (VHL); Cumulative Index to Nursing and Allied Health Literature (Cinahl), Embase, PubMed, Scopus and Web of Science Core Collection (WoS Core Collection). Subsequently, two catalogs of dissertations and theses were consulted to survey gray literature: one international, the Networked Digital Library of Theses and Dissertations (NDLTD), and another national, the *Biblioteca Digital Brasileira de Teses e Dissertações* (BDTD). Additionally, the reference lists of selected studies were consulted. 

 For the search strategy, descriptors combined with the Boolean operators “AND” and/or “OR” were used, as needed in each base. To select terms in English, the Medical Subject Headings (MeSH) was consulted, and in Portuguese, French and Spanish, the Health Sciences Descriptors (DeCS). Thus, composing the search strategy were the following terms: telenursing; remote consultation; patient cooperation; self-care; and chronic renal insufficiency ( Figure 1 ). The descriptor “conservative treatment” was not added to the search strategy, since its results capture studies that are not focused on its definition of prevention. 

**Figure 1:** - Search strategy implemented in the PubMed database. Niterói, RJ, Brazil, 2023

**Database**	**Strategy**
PubMed	((“Renal Insufficiency, Chronic”[mh] OR Chronic Kidney Disease*[tiab] OR Chronic Kidney Insufficiency*[tiab] OR Chronic Renal Disease*[tiab] OR Chronic Renal Insufficiency*[tiab]) AND ((“Remote Consultation”[mh] OR Remote Consultation[tiab] OR “Asynchronous Teleconsultation”[tiab] OR Teleconsultation[tiab] OR Telemonitoring[tiab] OR “Remote Monitoring”[tiab] OR “Remote Patient Monitoring”[tiab] OR Tele-Monitoring[tiab] OR Telehealth Monitoring[tiab] OR Telemedicine Monitoring[tiab] OR “Telemedicine”[mh] OR Telemedicine[tiab] OR “Mobile Health”[tiab] OR mHealth[tiab] OR Telehealth[tiab] OR eHealth[tiab] OR “Pervasive Health”[tiab] OR Telecare[tiab] OR Tele-Service*[tiab] OR Teleservices*[tiab] OR “Connected Health”[tiab] OR “Digital Health”[tiab] OR “telemedicine”[tiab] OR “virtual medicine”[tiab] OR “distant monitoring”[tiab] OR “distant patient monitoring”[tiab] OR “remote monitoring”[tiab] OR “remote patient monitoring”[tiab] OR “remote patient surveillance”[tiab] OR “tele monitoring”[tiab] OR “tele surveillance”[tiab] OR telesurveillance[tiab] OR “Telenursing”[mh] OR Telenursing[tiab] OR “Tele nursing”[tiab] OR tele-nursing[tiab] OR “virtual nursing”[tiab]))) AND ((“Self Care”[mh] OR “Self Care”[tiab] OR Self-Care[tiab] OR “Patient Compliance”[mh] OR “Patient Compliance”[tiab] OR Client Adherence*[tiab] OR Client Compliance*[tiab] OR Adherent Patient*[tiab] OR Patient Adherence*[tiab] OR Patient Cooperation[tiab] OR Patient Nonadherence[tiab] OR Patient Noncompliance[tiab] OR Therapeutic Compliance*[tiab] OR Treatment Compliance*[tiab] OR “Treatment Adherence and Compliance”[tiab] OR “Therapeutic Adherence”[tiab] OR “Treatment Adherence”[tiab] OR “Medication Adherence”[mh] OR Drug Adherence [tiab] OR Drug Compliance[tiab] OR Medication Compliance[tiab] OR Medication Adherence[tiab] OR Medication Nonadherence[tiab] OR Medication Noncompliance[tiab] OR Medication Persistence[tiab]))

It is noteworthy that in the VHL and BDTD it was necessary to divide the search strategy into two parts: one with descriptors in Portuguese, Spanish and French, and the other with descriptors only in English. This occurred because, when dealing with quadrilingual search strategies, the limit of searchable characters was reached.

### Selection of evidence sources

 After searching the databases and sources, the documents were selected, guided by the research question. The results obtained from the information sources were exported to the desktop version of the EndNote program to remove duplicate documents. Subsequently, they were added to the Rayyan software ^(^ ^15^ ^)^ , developed by the Qatar Computing Research Institute, enabling the selection and screening of studies, according to pre-defined criteria. The recommendations of the Prisma-ScR checklist were also considered ^(^ ^13^ ^)^ . 

 The selection of studies was carried out using the Rayyan software ^(^ ^15^ ^)^ , through double investigation (by peers), independently and blindly. In this way, titles and abstracts were first evaluated, observing whether they met the inclusion and exclusion criteria previously defined in the protocol. Those that met the requirements were read in full, and a new consensus meeting was held to resolve conflicts. 

### Data processing, analysis and extraction

After defining the sample, the selected studies had their content read thoroughly, in full, repeatedly, in order to allow data extraction and mapping.

Data extraction was carried out using a script prepared by the authors, giving rise to a database consisting of the characterization of publications and information relevant to the research object, containing: authorship/citation, year of publication, type of publication, language, country, study objective, study population, methodological design and main outcomes. To ensure compliance with the eligibility criteria, the script also included elements related to the PCC acronym, such as: GFR stage (P), interventions and technologies used associated with telenursing (C), and adherence (or non-adherence) to treatment and promotion of self-care in study results (C). The database was created using the Microsoft Excel program, and its content was filled in by one of the researchers and reviewed by their peer.

### Summary of results

In this research, the collected data was subjected to a narrative summary and descriptive statistical analysis. To present a clear and concise overview of the findings, the results were organized into tables and figures. This approach allowed for an in-depth understanding of the patterns and trends present in the study information, making it possible to both map them and consolidate the conclusions of this study.

### Ethical aspects

Regarding the ethical aspects of the research, there was no discrimination in the selection of articles or studies, respecting the individual blinding criterion.

## Results

**Figure 2:**
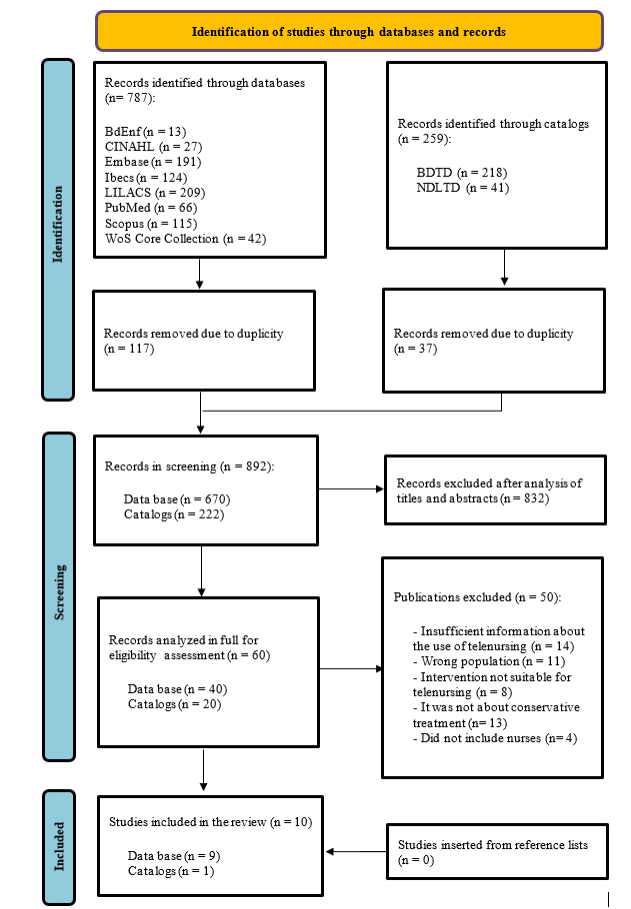
- PRISMA-ScR flowchart ^(^ ^13^ ^)^ . Niterói, RJ, Brazil, 2024

 The search identified a total of 1,046 potentially eligible studies in the information sources. These were exported to the desktop version of the EndNote program, from which it was possible to remove 154 duplicate studies. At the end of the screening in the Rayyan software ^(^ ^15^ ^)^ , a total of nine articles ^(^ ^16^ ^-^ ^24^ ^)^ and one dissertation ^(^ ^25^ ^)^ , developed in seven different countries, between the years 2011 and 2023, were selected to compose the scoping review. It is worth noting that, although the reference lists were consulted, it was not possible to capture studies that met the eligibility criteria. The study search and selection process was described in the Prisma-ScR flowchart ^(^ ^13^ ^)^ ( Figure 2 ). 

 According to Table 1 , regarding the characteristics of the ten selected studies, the first was published in 2011 and later in 2015, and the others were published discontinuously until 2023. The largest production occurred in 2019 and 2023, both with two articles. All studies are quantitative, with the majority being cohort or randomized controlled trials. About the language of publication, nine were published in English and one in Chinese. 

 Three studies had nurses as the focus of the intervention ^(^ ^16^ ^-^ ^17^ ^,^ ^25^ ^)^ . Four had their technologies developed by one or more nurses ^(^ ^19^ ^-^ ^22^ ^)^ . In other studies, the nurse was part of the multidisciplinary team, but maintained its relevance in the telehealth process ^(^ ^18^ ^,^ ^23^ ^-^ ^24^ ^)^ . Regarding the ways in which telehealth was used, the following were present: cell phone applications (60%), websites (20%), telephone support (10%) and teleconferencing (10%), as can be seen in the figure below ( Figure 3 ): 

**Table 1:** - Distribution of included studies according to country, type of study, approach and year of publication. Niterói, RJ, Brazil, 2023

**Characteristics**	**N** ^*^	**%** ^†^
**Country** Canada China United States of America Iran Ireland United Kingdom Taiwan	1 1 1 1 1 2 3	10 10 10 10 10 20 30
**Type of study** Randomized controlled trials Non-randomized controlled clinical trial Technological development research Cohort study Longitudinal feasibility study Pre-post test intervention study Observational study	2 1 1 3 1 1 1	20 10 10 30 1 1 1
**Approach** Quantitative	10	100
**Year of publication** 2011 2015 2016 2017 2019 2020 2021 2023	1 1 1 1 2 1 1 2	10 10 10 10 20 10 10 20
**GFR** ^§^ **stage** ^‡^ Stage 1 Stage 2 Stage 3 Stage 4 (non-dialysis) Stage 5 (non-dialysis) Not counted	1 0 5 1 2 1	10 0 50 10 20 10
**Adherence to treatment** Yes No Not counted	4 0 6	40 0 60
**Self-care** ^||^ Medication self-management Self-management of metabolic problems Knowledge of CKD ^¶^ Diet Exercise Smoking habit BP monitoring ^**^	8 1 7 6 6 1 2	80 10 70 60 60 10 20

^*^ n = Absolute frequency;

^†^ Relative frequency;

^§^ GFR = Glomerular filtration rate;

^‡^ Staging calculated from the average GFR, when not presented by the study. When present, the most prevalent stage was considered;

^||^ One or more items were indicated per study;

^¶^ CKD = Chronic kidney disease;

^**^ BP = Blood pressure

**Figure 3:** - Highlights of the main interventions. Niterói, RJ, Brazil, 2023

**Authorship**	**Interventions**	**Type of intervention**
Reston, et al. (2023) ^(^ ^16^ ^)^	CareKnowDo website. Website with 3 distinct modules: (1) Mind Matters: designed to deal with bad moods; (2) Lifestyle issues: mainly addressing diet, exercise and how these affect CKD ^*^ ; and (3) Medication Matters: covering adherence to antihypertensive medication. Each module included web-based activities and tools based on cognitive behavioral therapy and other evidence-based behavior change techniques. They also contained psychoeducational content designed to educate patients about CKD ^*^ and address key unhelpful beliefs that impact self-management behavior. Patients were also provided with a nursing line (telenursing) for questions or concerns.	Website
Liu, et al. (2023) ^(^ ^17^ ^)^	KidneyOnline intelligent system, developed for smartphones. Services provided: (1) Interpretation of the disease condition and corresponding guidance; (2) Regular check-ups; (3) Advance notices; (4) Real-time question and answer fields powered by knowledge graphs; and (5) Clinical reminders. It is a nurse-led, patient-oriented collaborative management system as a complement to regular clinical visits for patients with CKD ^*^ .	Mobile application
Tsai, et al. (2021) ^(^ ^18^ ^)^	Mobile application called iCKD, which has several key features, including home physiological testing, signal monitoring, health education about diseases and self-care, nutritional analysis, medication reminder and alarms, and an alert system. The application allows the multidisciplinary team (including nurses) to remotely analyze and monitor a patient’s health status with self-recorded data, enabling timely feedback to be sent with online reminders.	Mobile application
Winocour, et al. (2020) ^(^ ^19^ ^)^	Educational telehealth session via Skype, lasting two hours, aimed at reviewing virtual telehealth cases. To this end, a total of 20 clinical cases were pre-selected for discussion.	Conference call
Ellis, et al. (2019) ^(^ ^20^ ^)^	mHealth System: smart button device that self-monitors medication taking, companion smartphone application, computer algorithm used to determine adherence and, then, send standard or customized self-management support via text message (short message service) based on medication usage time. Standard self-management support text messages indicated that the smartphone application recorded the button press, while personalized self-management support text messages encouraged habit formation and systems thinking based on the time medications were taken.	Mobile application
Doyle, et al. (2019) ^(^ ^21^ ^)^	The MiKidney application records personal details, medical history, blood, weight and current medication list. It provides information about CKD ^*^ , CKD ^*^ medication, renal diet, kidney replacement treatment options, symptom management, and health maintenance. The application includes an exercise tracker and a daily log of exercises performed, reminder alerts and a notes section to record any queries or issues to be discussed with the disciplinary team. Additionally, the application has a “My Renal Rating” scoring scheme, with a traffic light system that provides feedback to users, including motivational messages.	Mobile application
Barahimi, et al. (2017) ^(^ ^22^ ^)^	E-learning as an educational intervention to improve kidney function and treat CKD ^*^ . The e-learning model used in the study was the ADDIE ^†^ , an acronym for analysis, design, development, implementation and evaluation. The content related to CKD ^*^ (definition, diagnosis, staging, risk factors, complications, care and monitoring) was identified using the “Kidney Disease: Improving Global Outcomes” guidelines ^(^ ^26^ ^)^ .	Website
Ong, et al. (2016) ^(^ ^23^ ^)^	Smartphone app-based self-management system designed and developed as a complement to usual CKD care ^*^ . The smartphone application targeted four behavioral elements: BP ^‡^ monitoring, medication management, symptom assessment, and laboratory results tracking. Email messages were sent automatically when responses required more urgent action, and recipients were determined by medical severity (e.g., nurse and/or pharmacist only or nurse, pharmacist and doctor).	Mobile application
Chen, et al. (2015) ^(^ ^24^ ^)^	Self-management support comprised health information, patient education, telephone support, and help from a support group. Telephone support involved a weekly phone call to improve CKD ^*^ self-management and ensure timely follow-up. The nurses were responsible for health education lectures, support groups and telephone support.	Telephone support
Lu (2011) ^(^ ^25^ ^)^	CKD ^*^ mobile health and self-care management system, developed for smartphones to help patients with self-monitoring of the disease. Nursing staff can monitor patients’ health status, send feedback and suggestions via instant messaging through smartphones, as well as search records and upload inspection reports via the web.	Mobile application

^*^ CKD = Chronic kidney disease;

^†^ ADDIE = Acronym for analysis, design, development, implementation and evaluation;

^‡^ BP = Blood pressure

 The studies surveyed in this review revealed that technological interventions contributed to improving the health of patients with CKD, through: improving disease monitoring ^(^ ^16^ ^-^ ^18^ ^,^ ^20^ ^-^ ^21^ ^,^ ^23^ ^,^ ^25^ ^)^ ; delaying renal progression ^(^ ^21^ ^,^ ^24^ ^)^ and reducing hospitalization events in advanced stages ^(^ ^24^ ^)^ ; that coordinated multidisciplinary care offered ideal patient management ^(^ ^18^ ^,^ ^24^ ^-^ ^25^ ^)^ and improved renal survival ^(^ ^24^ ^)^ ; the high level of acceptance and satisfaction by patients ^(^ ^23^ ^)^ ; signaling priority care for patients who need more attention ^(^ ^23^ ^)^ ; improvement in kidney function ^(^ ^19^ ^,^ ^21^ ^-^ ^22^ ^,^ ^24^ ^)^ ; better control of blood pressure ^(^ ^16^ ^-^ ^17^ ^,^ ^19^ ^)^ and glycemia ^(^ ^16^ ^,^ ^19^ ^)^ , weight reduction ^(^ ^19^ ^,^ ^21^ ^-^ ^22^ ^)^ and cardiorenal results ^(^ ^19^ ^)^ ; and reducing treatment costs, avoiding travel to the hospital or health unit ^(^ ^17^ ^,^ ^23^ ^)^ . 

## Discussion

### The use of telenursing technologies as adherence and self-care tools in the conservative treatment of CKD

 The consequences of CKD continue to pose enormous challenges around the world. Access and quality of care for CKD remain suboptimal in all settings, in part due to limited access to appropriate specialists to provide care ^(^ ^27^ ^)^ . By addressing these challenges, telenursing can extend people’s lifespan by optimizing operations, sharing interprofessional information, detecting health problems early, and treating them ^(^ ^28^ ^)^ . 

 The results show that the use of telenursing facilitates adherence to treatment and increases the patient’s ability to comply with medication regimens, due to self-monitoring ^(^ ^16^ ^-^ ^18^ ^,^ ^20^ ^-^ ^21^ ^,^ ^23^ ^-^ ^25^ ^)^ . Telenephrology promises to improve, increase or fill the gap in renal care in different countries, based on current levels of care, as demonstrated by a study carried out in the United States of America, whose adherence was relatively high in the sample due to the advantage of electronic monitoring ^(^ ^20^ ^)^ . 

 Also added is the expansion of knowledge about CKD ^(^ ^16^ ^-^ ^19^ ^,^ ^21^ ^-^ ^22^ ^,^ ^24^ ^)^ , since the effects of educational intervention establish the effectiveness of improving kidney function and treating the disease ^(^ ^22^ ^)^ . The use of e-learning, for example, is capable of generating a significant difference in terms of kidney function, and the use of the system in primary health care for patient education is recommended ^(^ ^22^ ^)^ . 

 Among the technologies associated with telehealth, the use of smartphone applications stood out ^(^ ^17^ ^-^ ^18^ ^,^ ^20^ ^-^ ^21^ ^,^ ^23^ ^,^ ^25^ ^)^ . They are able to simplify important tasks and support patient decision-making in real time, in addition to connecting them with their healthcare team without being intrusive, on either side, through a dynamic and customizable alert system ^(^ ^23^ ^)^ , increasing adherence to treatment ^(^ ^18^ ^,^ ^20^ ^,^ ^23^ ^-^ ^25^ ^)^ . 

 The use of websites ^(^ ^16^ ^,^ ^22^ ^)^ was also seen as a technology associated with telenursing, capable of promoting self-management and adherence to medication ^(^ ^16^ ^)^ , improving renal function ^(^ ^22^ ^)^ and treating CKD through educational intervention in the format e-learning ^(^ ^22^ ^)^ , acting as an easily accessible repository of information about CKD, so that patients can expand their understanding of their disease. 

 Other strategies for reducing the number of hospitalization events were the use of telephone support ^(^ ^24^ ^)^ and teleconferences ^(^ ^19^ ^)^ . In the case of telephone assistance, both individual and group, a dedicated space is guaranteed to provide information, support and the opportunity for individuals to express and share their experiences of living with CKD ^(^ ^29^ ^)^ . Its use has achieved great success in slowing the progression of diabetic and non-diabetic kidney disease. Multidisciplinary predialysis education decreased the incidence of dialysis and reduced all-cause mortality. Furthermore, after the establishment of CKD telecare and prevention centers, there was a drastic reduction in the incidence of end-stage renal insufficiency in Taiwan ^(^ ^24^ ^)^ . 

 In general, the studies that aimed to evaluate adherence ^(^ ^17^ ^-^ ^18^ ^,^ ^20^ ^,^ ^22^ ^)^ and self-care ^(^ ^16^ ^-^ ^25^ ^)^ showed an improvement in the analyzed indices, reinforcing that telenursing technologies were capable of influencing knowledge about CKD ^(^ ^16^ ^-^ ^19^ ^,^ ^21^ ^-^ ^22^ ^,^ ^24^ ^)^ , medication adherence ^(^ ^16^ ^-^ ^18^ ^,^ ^20^ ^-^ ^21^ ^,^ ^23^ ^-^ ^25^ ^)^ , diet ^(^ ^16^ ^-^ ^18^ ^,^ ^21^ ^,^ ^24^ ^-^ ^25^ ^)^ , blood pressure monitoring ^(^ ^18^ ^,^ ^23^ ^)^ , in the practice of exercises ^(^ ^16^ ^-^ ^18^ ^,^ ^21^ ^,^ ^24^ ^,^ ^25^ ^)^ and in lifestyle habits, such as smoking ^(^ ^18^ ^)^ . However, when it comes to knowledge about CKD, advanced age, low education and the duration of the disease influence the patient’s learning process ^(^ ^18^ ^)^ . 

 It is also worth highlighting that technologies that are easy to use and reach are useful mainly in patients with asymptomatic conditions ^(^ ^16^ ^)^ , in which the progression of the disease may go unnoticed due to the lack of perceptible symptoms, and be neglected. Therefore, this resource transmits to the patient information and knowledge necessary to understand CKD, its progression and severity, favoring adherence to treatment and their role in improving quality of life ^(^ ^16^ ^,^ ^20^ ^)^ . Furthermore, from a professional perspective, having a tool within reach that facilitates patient monitoring enables a more assertive and rapid intervention through constant vigilance, serving as support when doubts arise and providing support to the patient ^(^ ^16^ ^)^ . 

 Finally, it is important to consider the target population when using new technologies ^(^ ^16^ ^)^ , particularly when it involves the need for digital knowledge prior to their use, such as in the case of applications, websites or teleconferences. 

### The role of nursing in relation to telehealth technologies

 Telenursing is not restricted to teleconsultation and can be used to carry out clinical cases, staff management, team training and community service. It is especially useful for nurses who are not physically present, but whose guidance is critical to providing safe care, ensuring privacy and confidentiality in a comfortable environment ^(^ ^30^ ^)^ . 

 Among the results of the review, the nurse demonstrated to be a central part of telehealth practice. In studies in which telenursing was the focus ^(^ ^16^ ^-^ ^17^ ^,^ ^25^ ^)^ , their role as leader in technology management was essential for its implementation and effectiveness in the care of patients with CKD. Even in the multidisciplinary team, nurses maintain the relevance of their role in telehealth, providing support, guidance, education and monitoring of health status ^(^ ^18^ ^,^ ^23^ ^-^ ^24^ ^)^ . 

 The use of telenursing facilitates the implantation of guidelines and training among caregivers ^(^ ^27^ ^)^ and provides patient education and awareness about active involvement in their own care, offering best practices for engaging in self-care (self-management) ^(^ ^22^ ^,^ ^25^ ^)^ . It is revealed as a research, care and management practice, which can promote adherence, improve access to care and patient safety, as well as create an interprofessional communication and information network, encouraging this new practice and the production and technological innovation ^(^ ^28^ ^)^ . 

 Therefore, it is beneficial for nurses to know their patient and understand that, despite all the changes that the disease brings, there is a life beyond CKD. The nurse also needs to take care of their own values and attitudes when creating a mutual relationship with the individual ^(^ ^29^ ^)^ . 

 It is noteworthy that the implications of raising awareness of patient involvement, supporting care practices that promote their engagement and empowerment, in addition to contributing to the reflection of the renal patient’s attitude towards surveillance and control of their own treatment, promote health care, as a result of the partnership between professionals and users, in order to contribute to breaking the status of the “passive patient”, underlying the disease-centered care model, so present in health services ^(^ ^31^ ^)^ . 

 In short, given the potential for remote care, remote work presents itself as an innovation in nursing practice and in the health area, at the same time as a challenge for professionals ^(^ ^32^ ^)^ , which provides opportunities for better organization of the health service in a crisis situation, as occurred during the COVID-19 pandemic, allowing those involved in patient care to assist them safely and with quality. 

 Considering that telenursing is still a developing practice in several countries ^(^ ^33^ ^)^ , and that most studies did not have a longitudinal time frame, it is possible to observe as a knowledge gap the lack of long-term results from the implementation of teleconsultation in regression or stabilization of CKD, mainly when we consider the use of technological innovations, principally in the early stages of the disease. 

## Conclusion

It was evident that the use of telehealth by nursing is relevant in the context of health, both in social and economic terms, particularly considering the severity of CKD as a global health condition. Its use is capable of reducing the early progression of kidney function loss, but it is still little explored in national and international literature, representing a gap in knowledge to be filled in future research. Studies have shown that its implementation helps and supports health professionals, providing inferences and guidance on quick, safe and effective assistance, even remotely, through platforms, teleconferences, telemessaging and health technology service portals.

Acting as a means of preventing and promoting health remotely, telenursing presents itself as a strategy capable of promoting adherence to treatment and self-care in patients with chronic renal insufficiency undergoing conservative treatment. It favors the improvement of clinical practice and knowledge, and the development of health technologies and innovation in the field of nursing, contributing to scientific advancement and the development of public policies in the area of chronic non-communicable diseases, and can therefore generate a positive impact on public health.

Finally, given the gaps identified, it is recommended that future studies be carried out to monitor the use of health technologies to improve self-care and adherence to treatment in patients with CKD, from their initial pre-dialysis stages to entry into renal replacement therapy.
