# Supplementary material for: Use of Telenursing in the conservative treatment of patients with chronic renal insufficiency: scoping review
Source: Rev Lat Am Enfermagem. 2024 Oct 25;32:e4359. doi: 10.1590/1518-8345.7013.4359 (PMC11526289; doi:10.1590/1518-8345.7013.4359)
Supplement: Translation

# Translation: Uso de la Teleenfermería en el tratamiento conservador de pacientes con insuficiencia renal crónica: revisión de alcance ^*^

**DOI:** 10.1590/1518-8345.7013.4358

**Authors:** Aline de Oliveira Biancamano, Alessandra Conceição Leite Funchal Camacho, Elaine Antunes Cortez, Harlon França de Menezes, Yasmin Saba de Almeida, Cristiele Costa da Matta Rocha

**Affiliations:** ^1^ Universidade Federal Fluminense, Escola de Enfermagem Aurora de Afonso Costa, Niterói, RJ, Brasil.; ^2^ Ministério da Saúde, Hospital Federal dos Servidores do Estado, Serviço de Nefrologia, Rio de Janeiro, RJ, Brasil.; ^3^ Universidade Federal Fluminense, Escola de Enfermagem Aurora de Afonso Costa, Departamento de Fundamentos de Enfermagem e Administração, Niterói, RJ, Brasil.; ^4^ Universidade Federal Fluminense, Escola de Enfermagem Aurora de Afonso Costa, Departamento de Enfermagem Materno-Infantil e Psiquiátrica, Niterói, RJ, Brasil.

**Keywords:** Teleenfermería, Cumplimiento y Adherencia al Tratamiento, Autocuidado, Insuficiencia Renal Crónica, Tratamiento Conservador, Tecnología Biomédica

## Abstract

**Objetivo:**

mapear la evidencia del uso de la teleenfermería en la adherencia al tratamiento y promoción del autocuidado en pacientes con insuficiencia renal crónica en tratamiento conservador.

**Método:**

estudio de revisión de alcance, con selección de documentos indexados en ocho bases de datos y dos catálogos de tesis y disertaciones. Además, se consultaron las listas de referencias de los estudios seleccionados. La selección y análisis de los estudios se realizó mediante el *software* *Rayyan* por medio investigación doble, independiente y ciega.

**Resultados:**

se encontraron 56 publicaciones elegibles y se seleccionaron diez para componer el estudio. Entre las intervenciones de teleenfermería se destacaron: aplicaciones para celulares, sitios *web* , plataforma digital, atención telefónica y teleconferencias.

**Conclusión:**

se evidenció que el uso de la Telesalud por parte de Enfermería es relevante para el sector de la salud, pero aún poco explorado en la literatura nacional e internacional, representando un vacío de conocimiento a ser llenado en futuras investigaciones. Estudios han demostrado que su implementación ayuda y apoya a los profesionales de la salud, brindando inferencias y orientaciones para una asistencia rápida, segura y eficaz, incluso a distancia. La teleenfermería se presenta como una estrategia capaz de promover la adherencia al tratamiento y al autocuidado en pacientes con insuficiencia renal crónica en tratamiento conservador.

## Puntos destacados

**(1)** Desarrolla estrategias de innovación en salud para el tratamiento remoto.

**(2)** Ofrece apoyo a los profesionales con medidas preventivas, minimizando costos.

**(3)** Estimula la práctica de la autogestión del cuidado.

**(4)** Proporciona inferencias y orientaciones para una asistencia rápida y segura.

**(5)** Contribuye al avance de políticas públicas en materia de enfermedades no transmisibles.

## Introducción

 La insuficiencia renal crónica se define por la presencia de cambios en la estructura o funciones de los riñones, con o sin alteración en la filtración glomerular, por un período superior a tres meses ^(^ ^1^ ^)^ . Es la tasa de filtración glomerular (TFG) la que mayor correlación tiene con los resultados clínicos, a pesar de que la creatinina sea el mejor marcador de la función renal ^(^ ^2^ ^)^ . El nivel de TFG varía según la edad, el género y la masa muscular. A medida que avanza la edad, es posible observar una disminución de la TFG, y esta reducción puede manifestarse mucho antes de la aparición de los síntomas y estar asociada con la gravedad de la enfermedad renal crónica (ERC) ^(^ ^3^ ^)^ . 

 La ERC es progresiva e irreversible hasta la fecha, pero puede tratarse inicialmente mediante medidas terapéuticas conservadoras, que consisten en medidas clínicas utilizadas para retrasar el empeoramiento de la función renal, reducir los síntomas y prevenir complicaciones ^(^ ^4^ ^)^ . Iniciar tempranamente el tratamiento conservador aumenta las posibilidades de preservar la función renal durante más tiempo ^(^ ^5^ ^)^ . 

 El tratamiento conservador aborda el manejo de los factores de riesgo para prevenir la progresión de la enfermedad renal en sus etapas iniciales, que van de 1 a 3, cuando la TFG se encuentra entre ≥90 y 30 mL/min/1,73 m ^2^ . También engloba la fase prediálisis en las etapas 4 y 5, no dialíticas, cuando la TFG varía de 29 a <15 mL/min/1,73 m ^2^ , e incluye la terapia de reemplazo renal cuando se alcanza la etapa 5 dialítica, en la que la TFG ya está por debajo de 15 mL/min/1,73 m ^2^^(^ ^2^ ^)^ . 

 El control de la ERC por parte del paciente está directamente relacionado con los cambios en los hábitos de vida, y estos se consiguen eficazmente cuando se incluyen en las actividades de cuidado del día a día que la persona realiza para mejorar su propia salud (autocuidado) ^(^ ^6^ ^)^ . 

 Debido a que las primeras etapas de la ERC suelen ser asintomáticas y fácilmente ignoradas, el conocimiento de la enfermedad es bajo y los pacientes a menudo retrasan y descuidan el tratamiento. Por ello, los profesionales de la salud que atienden a usuarios con enfermedades crónicas necesitan nuevos modelos que les ayuden a desarrollar habilidades comunicativas avanzadas ^(^ ^3^ ^)^ . 

 Con la llegada de la pandemia y la necesidad de mantener el aislamiento y el distanciamiento social, el Consejo Federal de Enfermería (Cofen), en 2020 ^(^ ^7^ ^)^ , decidió estandarizar la práctica de la teleconsulta de enfermería en Brasil y, posteriormente, en 2022, creó la Resolución nº 696/2022 ^(^ ^8^ ^)^ , estandarizando la teleenfermería con el papel del enfermero en la salud digital. La teleenfermería integra la telesalud y se caracteriza por el uso de recursos tecnológicos para realizar la práctica de enfermería de forma remota, en las dimensiones: asistencial, educativa o de investigación ^(^ ^9^ ^)^ . 

 Desde esa perspectiva, una revisión integradora ^(^ ^10^ ^)^ evaluó el uso de la teleenfermería en el cuidado de pacientes crónicos y demostró que esa tecnología brindó beneficios a los pacientes, a los profesionales y al sistema de salud. Respecto a los pacientes, hubo una sensación de empoderamiento, mejor manejo de la enfermedad, disminución de la ansiedad, mejor calidad de vida y mayor adherencia a la medicación. En cuanto a los profesionales, hubo un aumento de la comunicación y del apoyo, un mejor manejo de los síntomas, lo que permitió diagnóstico e intervención precoces, y un ahorro de tiempo de trabajo. Finalmente, para el sistema de salud, se detectó una reducción en la tasa de ingresos hospitalarios y consultas ambulatorias. 

 Entendiendo el potencial de la teleenfermería para el cuidado de pacientes con ERC y la urgencia de iniciar un tratamiento conservador, los estudios que buscan retratar su uso se vuelven esenciales para la salud pública. Sin embargo, la búsqueda de una comprensión más profunda del tema sigue siendo un desafío, ya que hay escasez de materiales debido a lo novedoso de la práctica, y la poca literatura disponible está dispersa o es de difícil acceso. Por lo tanto, esta revisión de alcance se basa en la inminencia de brindar visibilidad y comprensión al concepto central del estudio, respaldada por la brecha de conocimiento identificada por medio de búsquedas previas en *Prospero* , *Cochrane Database of Systematic Reviews* y *JBI Evidence Synthesis* , cuyos resultados demuestran que no hay revisiones, completadas o en progreso, con este enfoque. 

Por lo tanto, se justifica esta revisión de alcance, que tiene como objetivo mapear la evidencia del uso de la teleenfermería en la adherencia al tratamiento y la promoción del autocuidado en pacientes con insuficiencia renal crónica sometidos a tratamiento conservador. Este estudio puede contribuir a la difusión de un nuevo enfoque clínico-práctico para la promoción de la salud en el tratamiento conservador en pacientes con insuficiencia renal crónica.

## Método

### Diseño del estudio

 Este es un estudio de revisión de alcance, según el método de revisión propuesto por la colaboración JBI ^(^ ^11^ ^)^ . Este método permite proporcionar una síntesis completa e imparcial de estudios relevantes dentro de los límites de un solo documento, utilizando métodos rigurosos y transparentes ^(^ ^12^ ^)^ . Para informar la revisión, se utilizó el *Preferred Reporting Items for Systematic reviews and Meta-Analyses extension for Scoping Reviews* (Prisma-ScR) *checklist* ^(^ ^13^ ^)^ . 

Por lo tanto, el objetivo principal de esta investigación es mapear la evidencia sobre el uso de la teleenfermería, guiada por la siguiente pregunta: ¿Cómo influye el uso de la teleenfermería en la adherencia al tratamiento y la promoción del autocuidado en pacientes con insuficiencia renal crónica en tratamiento conservador? Para construir la pregunta de investigación se utilizó la estrategia mnemotécnica población, concepto y contexto (PCC). Se definieron: población - personas con insuficiencia renal crónica en tratamiento conservador; concepto - teleenfermería; y contexto - adherencia al tratamiento y promoción del autocuidado.

### Protocolo y registro

 Inicialmente se realizó un protocolo de investigación con el objetivo de garantizar el rigor metodológico de la revisión, y sus componentes comprendieron las siguientes fases: definición del objetivo y pregunta de investigación; definición de los criterios de elegibilidad (inclusión y exclusión); planificación de la estrategia de búsqueda y selección de los estudios con relación a los descriptores específicos de cada base de datos; identificación de los estudios y selección de los estudios; extracción de los datos; mapeo de los datos; y resumen de los resultados. El protocolo final fue registrado en la plataforma *Open Science Framework* (OSF) ^(^ ^14^ ^)^ . 

### Criterios de elegibilidad

De acuerdo con el acrónimo PCC, se establecieron los siguientes criterios de elegibilidad: inclusión - estudios primarios transversales, observacionales, ensayos clínicos, estudios cuasiexperimentales y randomizados, realizados con pacientes adultos (18 años o más), publicados en cualquier idioma, sin delimitación de período; exclusión - cartas al editor y editoriales; estudios que solamente abordaran la insuficiencia renal crónica en el tratamiento conservador, pero no abordaran la teleenfermería; estudios que abordaran la terapia dialítica sin tratamiento conservador como uno de los métodos o método exclusivo; estudios que no contaran con al menos un enfermero profesional trabajando o desarrollando la tecnología asociada con la telesalud; y, además, que el abordaje no fuera un dispositivo de telesalud.

Es importante señalar que, aunque inicialmente se consideraron estudios secundarios para su inclusión en la revisión, ninguna publicación con este diseño respondió a la pregunta de investigación.

### Fuentes de información e investigación

 La búsqueda se realizó el 6 de enero de 2023 y se actualizó el 23 de abril de 2024. En primer lugar, se consultaron las siguientes bases de datos electrónicas: Base de Datos de Enfermería (BdEnf), Índice Bibliográfico Español en Ciencias de la Salud (Ibecs) y Literatura Latinoamericana y del Caribe en Ciencias de la Salud (LILACS), por medio de la Biblioteca Virtual en Salud (BVS); *Cumulative Index to Nursing and Allied Health Literature* (CINAHL), Embase, PubMed, *Scopus* y *Web of Science Core Collection* ( *WoS Core Collection* ). Posteriormente, se consultaron dos catálogos de disertaciones y tesis para relevar la literatura gris: uno internacional, la *Networked Digital Library of Theses and Dissertations* (NDLTD), y otro nacional, la *Biblioteca Digital Brasileira de Teses e Dissertações* (BDTD). Además, se consultaron las listas de referencias de los estudios seleccionados. 

 Para la estrategia de búsqueda se utilizaron descriptores combinados con los operadores booleanos “AND” y/u “OR”, según fuera necesario en cada base. Para seleccionar términos en inglés se consultó el *Medical Subject Headings* (MeSH), y en portugués, francés y español, los Descriptores en Ciencias de la Salud (DeCS). Así, compusieron la estrategia de búsqueda los siguientes términos: teleenfermería; consulta remota; cooperación del paciente; autocuidado; e insuficiencia renal crónica ( Figura 1 ). El descriptor “tratamiento conservador” no se agregó a la estrategia de búsqueda, ya que sus resultados captan estudios que no se centran en su definición de prevención. 

**Figura 1:** - Estrategia de búsqueda implementada en la base de datos PubMed. Niterói, RJ, Brasil, 2023

**Bases de datos**	**Estrategia**
PubMed	((“Renal Insufficiency, Chronic”[mh] OR Chronic Kidney Disease ^*^ [tiab] OR Chronic Kidney Insufficiency ^*^ [tiab] OR Chronic Renal Disease ^*^ [tiab] OR Chronic Renal Insufficiency ^*^ [tiab]) AND ((“Remote Consultation”[mh] OR Remote Consultation[tiab] OR “Asynchronous Teleconsultation”[tiab] OR Teleconsultation[tiab] OR Telemonitoring[tiab] OR “Remote Monitoring”[tiab] OR “Remote Patient Monitoring”[tiab] OR Tele-Monitoring[tiab] OR Telehealth Monitoring[tiab] OR Telemedicine Monitoring[tiab] OR “Telemedicine”[mh] OR Telemedicine[tiab] OR “Mobile Health”[tiab] OR mHealth[tiab] OR Telehealth[tiab] OR eHealth[tiab] OR “Pervasive Health”[tiab] OR Telecare[tiab] OR Tele-Service ^*^ [tiab] OR Teleservices ^*^ [tiab] OR “Connected Health”[tiab] OR “Digital Health”[tiab] OR “telemedicine”[tiab] OR “virtual medicine”[tiab] OR “distant monitoring”[tiab] OR “distant patient monitoring”[tiab] OR “remote monitoring”[tiab] OR “remote patient monitoring”[tiab] OR “remote patient surveillance”[tiab] OR “tele monitoring”[tiab] OR “tele surveillance”[tiab] OR telesurveillance[tiab] OR “Telenursing”[mh] OR Telenursing[tiab] OR “Tele nursing”[tiab] OR tele-nursing[tiab] OR “virtual nursing”[tiab]))) AND ((“Self Care”[mh] OR “Self Care”[tiab] OR Self-Care[tiab] OR “Patient Compliance”[mh] OR “Patient Compliance”[tiab] OR Client Adherence ^*^ [tiab] OR Client Compliance ^*^ [tiab] OR Adherent Patient ^*^ [tiab] OR Patient Adherence ^*^ [tiab] OR Patient Cooperation[tiab] OR Patient Nonadherence[tiab] OR Patient Noncompliance[tiab] OR Therapeutic Compliance ^*^ [tiab] OR Treatment Compliance ^*^ [tiab] OR “Treatment Adherence and Compliance”[tiab] OR “Therapeutic Adherence”[tiab] OR “Treatment Adherence”[tiab] OR “Medication Adherence”[mh] OR Drug Adherence [tiab] OR Drug Compliance[tiab] OR Medication Compliance[tiab] OR Medication Adherence[tiab] OR Medication Nonadherence[tiab] OR Medication Noncompliance[tiab] OR Medication Persistence[tiab]))

Se destaca que en la BVS y en la BDTD fue necesario dividir la estrategia de búsqueda en dos partes: una con descriptores en portugués, español y francés, y otra con descriptores solamente en inglés. Esto ocurrió porque, al tratarse de estrategias de búsqueda cuatrilingües, se alcanzó el límite de caracteres buscables.

### Selección de las fuentes de evidencia

 Luego de buscar en las bases de datos y fuentes, se seleccionaron los documentos, guiados por la pregunta de investigación. Los resultados obtenidos de las fuentes de información se exportaron a la versión *desktop* del programa *EndNote* para eliminar documentos duplicados. Posteriormente, se agregaron al *software* *Rayyan* ^(^ ^15^ ^)^ , desarrollado por el *Qatar Computing Research Institute* , que permitió la selección y triaje de los estudios, según criterios predefinidos. También se consideraron las recomendaciones del Prisma-ScR *checklist* ^(^ ^13^ ^)^ . 

 La selección de los estudios se realizó mediante el *software Rayyan* ^(^ ^15^ ^)^ , por medio de investigación doble (por pares), de forma independiente y ciega. De este modo, en primer lugar se evaluaron los títulos y resúmenes, observando si cumplían con los criterios de inclusión y exclusión previamente definidos en el protocolo. Aquellos que cumplieron con los requisitos fueron leídos en su totalidad, y se realizó una nueva reunión de consenso para resolver los conflictos. 

### Procesamiento, análisis y extracción de datos

Después de definir la muestra, se leyó detenidamente el contenido de los estudios seleccionados, en su totalidad y repetidamente, para permitir la extracción y el mapeo de datos.

 La extracción de datos se realizó mediante un guion elaborado por los autores, dando lugar a una base de datos compuesta por la caracterización de publicaciones e información relevante al objeto de investigación, que contiene: autoría/cita, año de publicación, tipo de publicación, idioma, país, objetivo del estudio, población del estudio, diseño metodológico y resultados principales. Para garantizar el cumplimiento de los criterios de elegibilidad, el guión también incluyó elementos relacionados con el acrónimo PCC, tales como: etapa de la TFG (P), intervenciones y tecnologías utilizadas asociadas a la teleenfermería (C), y adherencia (o no adherencia) al tratamiento y promoción del autocuidado en los resultados del estudio (C). La base de datos fue creada utilizando el programa *Microsoft Excel* , y su contenido fue completado por uno de los investigadores y revisado por su par. 

### Síntesis de los resultados

En esta investigación, los datos recolectados fueron sometidos a un resumen narrativo y análisis estadístico descriptivo. Para presentar una descripción general clara y concisa de los hallazgos, los resultados se organizaron en tablas y figuras. Este enfoque permitió comprender en profundidad los patrones y tendencias presentes en la información del estudio, permitiendo mapearlos y consolidar las conclusiones de este estudio.

### Aspectos éticos

En cuanto a los aspectos éticos de la investigación, no hubo discriminación en la selección de artículos o estudios, respetando el criterio de cegamiento individual.

## Resultados

 La búsqueda identificó un total de 1.046 estudios potencialmente elegibles en las fuentes de información. Estos fueron exportados a la versión *desktop* del programa *EndNote* , desde donde fue posible eliminar 154 estudios duplicados. Al final del triaje en el *software Rayyan* ^(^ ^15^ ^)^ , se seleccionaron un total de nueve artículos ^(^ ^16^ ^-^ ^24^ ^)^ y una disertación ^(^ ^25^ ^)^ , desarrollados en siete países diferentes, entre los años 2011 y 2023, para componer la revisión de alcance. Cabe señalar que, aunque se consultaron las listas de referencias, no fue posible capturar estudios que cumplieran con los criterios de elegibilidad. El proceso de búsqueda y selección de estudios se describió en el flujograma Prisma-ScR ^(^ ^13^ ^)^ ( Figura 2 ). 

 Según la Tabla 1 , en cuanto a las características de los diez estudios seleccionados, el primero fue publicado en 2011 y posteriormente en 2015, y los demás se publicaron de forma discontinua hasta 2023. La mayor producción se produjo en 2019 y 2023, ambos con dos artículos. Todos los estudios son cuantitativos, y la mayoría son ensayos controlados randomizados o de cohortes. En cuanto al idioma de publicación, nueve se publicaron en inglés y uno en chino. 

 Tres estudios tuvieron al enfermero como foco de la intervención ^(^ ^16^ ^-^ ^17^ ^,^ ^25^ ^)^ . Cuatro tuvieron sus tecnologías desarrolladas por uno o más enfermeros ^(^ ^19^ ^-^ ^22^ ^)^ . En otros estudios, el enfermero formó parte del equipo multidisciplinario, pero mantuvo su relevancia en el proceso de telesalud ^(^ ^18^ ^,^ ^23^ ^-^ ^24^ ^)^ . En cuanto a las formas en que se utilizó la telesalud, estuvieron presentes: aplicaciones de celular (60%), sitios *web* (20%), atención telefónica (10%) y teleconferencias (10%), como se puede observar en la siguiente figura ( Figura 3 ). 

**Figura 2:** - Flujograma PRISMA-ScR ^(^ ^13^ ^)^ . Niterói, RJ, Brasil, 2024

**Tabla 1:** - Distribución de los estudios incluidos según país, tipo de estudio, enfoque y año de publicación. Niterói, RJ, Brasil, 2023

**Características**	**N** ^*^	**%** ^†^
**País** Canadá China Estados Unidos de América Irán Irlanda Reino Unido Taiwán	1 1 1 1 1 2 3	10 10 10 10 10 20 30
**Tipo de estudio** Ensayos controlados randomizados Ensayo clínico controlado no randomizado Investigaciones de desarrollo tecnológico Estudio de cohorte Estudio de viabilidad longitudinal Estudio de intervención pre-post test Estudio observacional	2 1 1 3 1 1 1	20 10 10 30 1 1 1
**Enfoque** Cuantitativo	10	100
**Año de publicación** 2011 2015 2016 2017 2019 2020 2021 2023	1 1 1 1 2 1 1 2	10 10 10 10 20 10 10 20
**Etapa** ^‡^ **de la TFG** ^§^ Etapa 1 Etapa 2 Etapa 3 Etapa 4 (no dialítica) Etapa 5 (no dialítica) No contabilizado	1 0 5 1 2 1	10 0 50 10 20 10
**Adherencia al tratamiento** Sí No No contabilizado	4 0 6	40 0 60
**Autocuidado** ^||^ Autogestión de medicamentos Autogestión de problemas metabólicos Conocimiento de la ERC ^¶^ Dieta Ejercicio Hábito de fumar Monitoreo de la PA ^**^	8 1 7 6 6 1 2	80 10 70 60 60 10 20

^*^ n = Frecuencia absoluta;

^†^ Frecuencia relativa;

^‡^ Estadificación calculada a partir de la TFG promedio, cuando no lo presentaba el estudio. Cuando estaba presente, se consideraba la etapa más prevalente;

^§^ TFG = Tasa de filtración glomerular;

^||^ Hubo indicación de uno o más ítems por estudio;

^¶^ ERC = Enfermedad renal crónica;

^**^ PA = Presión arterial

**Figura 3:** - Destaques de las principales intervenciones. Niterói, RJ, Brasil, 2023

**Autoría**	**Intervenciones**	**Tipo de intervención**
Reston, et al. (2023) ^(^ ^16^ ^)^	Sitio *web* *CareKnowDo* . Sitio *web* con 3 módulos distintos: (1) *Mind Matters* : diseñado para lidiar con el mal humor; (2) Cuestiones de estilo de vida: abordando principalmente la dieta, el ejercicio y cómo estos afectan la ERC ^*^ ; y (3) Asuntos de Medicación: cubriendo la adherencia a la medicación antihipertensiva. Cada módulo incluía actividades y herramientas basadas en internet y en la terapia cognitivo-conductual y otras técnicas de cambio de comportamiento basadas en evidencia. También tenían contenido psicoeducativo diseñado para educar a los pacientes sobre la ERC ^*^ y abordar las principales creencias inútiles que afectan el comportamiento de autocuidado. Los pacientes también contaron con una línea de enfermería (teleenfermería) para preguntas o inquietudes.	Sitio *web*
Liu, et al. (2023) ^(^ ^17^ ^)^	Sistema inteligente *KidneyOnline* , desarrollado para *smartphones* . Servicios prestados: (1) Interpretación del estado de la enfermedad y orientación correspondiente; (2) *Check-ups* regulares; (3) Avisos anticipados; (4) Campos de preguntas y respuestas en tiempo real impulsados por gráficos de conocimiento; y (5) Recordatorios clínicos. Es un sistema de gestión colaborativa dirigido por enfermeros y orientado al paciente como complemento a las visitas clínicas regulares para pacientes con ERC ^*^ .	Aplicación móvil
Tsai, et al. (2021) ^(^ ^18^ ^)^	Aplicación móvil denominada iCKD, que cuenta con varios recursos principales, que incluyen pruebas fisiológicas en el hogar, monitoreo de señales, educación en salud sobre enfermedades y autocuidado, análisis nutricional, recordatorios y alarmas de medicación, y un sistema de alerta. La aplicación permite al equipo multidisciplinario (incluido el enfermero) analizar y monitorear de forma remota el estado de salud de un paciente con datos autoregistrados, lo que posibilita enviar *feedbacks* oportunos con recordatorios *on line* .	Aplicación móvil
Winocour, et al. (2020) ^(^ ^19^ ^)^	Sesión educativa de telesalud vía *Skype* , de dos horas de duración, orientada a la revisión de casos de telesalud virtual. Para ello, se preseleccionaron un total de 20 casos clínicos para su discusión.	Teleconferencia
Ellis, et al. (2019) ^(^ ^20^ ^)^	Sistema *mHealth* : dispositivo de botón inteligente que autocontrola la toma de medicamentos, aplicación complementaria para *smartphone* , algoritmo informático utilizado para determinar la adherencia y luego enviar apoyo de autogestión ( *self-management support* ) estándar o personalizado a través de mensajes de texto (servicio de mensajes cortos) según el horario de uso del medicamento. Los mensajes de texto *self-management support* estándar indicaban que la aplicación para *smartphone* registró la pulsación del botón, mientras que los mensajes de texto *self-management support* personalizados fomentaban la formación de hábitos y el pensamiento sistémico basado en el horario en que se tomaban los medicamentos.	Aplicación móvil
Doyle, et al. (2019) ^(^ ^21^ ^)^	La aplicación *MiKidney* registra datos personales, historial médico, sangre, peso y lista de medicamentos actuales. Proporciona información sobre ERC ^*^ , medicamentos para ERC ^*^ , dieta renal, opciones de tratamiento de reemplazo renal, manejo de los síntomas, y mantenimiento de la salud. La aplicación incluye un rastreador de ejercicios y un registro diario de los ejercicios realizados, alertas recordatorias y una sección de notas para registrar cualquier consulta o problema que se deba discutir con el equipo disciplinario. Además, la aplicación cuenta con un esquema de puntuación “ *My Renal Rating* ”, con un sistema de semáforo que proporciona *feedback* a los usuarios, incluidos mensajes motivadores.	Aplicación móvil
Barahimi, et al. (2017) ^(^ ^22^ ^)^	*E-learning* como intervención educativa para mejorar la función renal y tratar la ERC ^*^ . El modelo de *e-learning* utilizado en el estudio fue el modelo ADDIE ^†^ , acrónimo de análisis, diseño, desarrollo, implementación y evaluación. El contenido relacionado con la ERC ^*^ (definición, diagnóstico, estadificación, factores de riesgo, complicaciones, cuidados y seguimiento) se identificó utilizando las *guidelines* del “ *Kidney Disease: Improving Global Outcomes* ” ^(^ ^26^ ^)^ .	Sitio *web*
Ong, et al. (2016) ^(^ ^23^ ^)^	Sistema de autogestión basado en aplicación para *smartphone* , diseñado y desarrollado como complemento a los cuidados habituales de la ERC ^*^ . La aplicación para *smartphone* se centró en cuatro elementos comportamentales: monitorización de la PA ^‡^ , gestión de la medicación, evaluación de los síntomas, y seguimiento de los resultados de laboratorio. Los mensajes de correo electrónico se enviaban automáticamente cuando las respuestas requerían acciones más urgentes, y los destinatarios se determinaban según la gravedad médica (p. ej., enfermero y/o farmacéutico únicamente o enfermero, farmacéutico y médico).	Aplicación móvil
Chen, et al. (2015) ^(^ ^24^ ^)^	El apoyo a la autogestión comprendía información de salud, educación del paciente, asistencia telefónica y asistencia de un grupo de apoyo. La asistencia telefónica implicó una llamada telefónica semanal para mejorar la autogestión de la ERC ^*^ y garantizar un seguimiento oportuno. Los enfermeros fueron responsables de conferencias de educación en salud, grupos de apoyo y atención telefónica.	Atención telefónica
Lu (2011) ^(^ ^25^ ^)^	Sistema móvil de gestión de salud y autocuidado de ERC ^*^ , desarrollado para *smartphones* para ayudar a los pacientes con el autocontrol de la enfermedad. El personal de enfermería puede monitorear el estado de salud de los pacientes, enviar *feedbacks* y sugerencias vía mensajería instantánea a través de los *smartphones* , así como buscar registros y cargar informes de inspección por la *web* .	Aplicación móvil

^*^ ERC = Enfermedad renal crónica;

^†^ ADDIE = Acrónimo de análisis, diseño, desarrollo, implementación y evaluación.;

^‡^ PA = Presión arterial

 Los estudios relevados en esta revisión evidenciaron que las intervenciones tecnológicas contribuyeron a mejorar la salud de los pacientes con ERC, por medio de: mejora del seguimiento de la enfermedad ^(^ ^16^ ^-^ ^18^ ^,^ ^20^ ^-^ ^21^ ^,^ ^23^ ^,^ ^25^ ^)^ ; retraso de la progresión renal ^(^ ^21^ ^,^ ^24^ ^)^ y reducción de los eventos de hospitalización en etapa avanzada ^(^ ^24^ ^)^ ; que la atención multidisciplinaria coordinada ofreció un manejo ideal del paciente ^(^ ^18^ ^,^ ^24^ ^-^ ^25^ ^)^ y mejoró la supervivencia renal ^(^ ^24^ ^)^ ; alto nivel de aceptación y satisfacción por parte de los pacientes ^(^ ^23^ ^)^ ; señalización de la atención prioritaria a los pacientes que necesitan más atención ^(^ ^23^ ^)^ ; mejora de la función renal ^(^ ^19^ ^,^ ^21^ ^-^ ^22^ ^,^ ^24^ ^)^ ; mejor control de la presión arterial ^(^ ^16^ ^-^ ^17^ ^,^ ^19^ ^)^ y glucemia ^(^ ^16^ ^,^ ^19^ ^)^ , reducción de peso ^(^ ^19^ ^,^ ^21^ ^-^ ^22^ ^)^ y resultado cardiorrenal ^(^ ^19^ ^)^ ; y reducción de los costos de tratamiento, evitando desplazamientos al hospital o unidad de salud ^(^ ^17^ ^,^ ^23^ ^)^ . 

## Discusión

### El uso de tecnologías de teleenfermería como herramientas de adherencia y autocuidado en el tratamiento conservador de la ERC

 Las consecuencias de la ERC siguen planteando enormes desafíos en todo el mundo. El acceso y la calidad de los cuidados para la ERC siguen siendo subóptimos en todos los entornos, en parte debido al acceso limitado a especialistas adecuados para brindar atención ^(^ ^27^ ^)^ . Al abordar estos desafíos, la teleenfermería puede extender la vida útil de las personas al optimizar las operaciones, compartir información interprofesional, detectar tempranamente problemas de salud y tratarlos ^(^ ^28^ ^)^ . 

 Los resultados muestran que el uso de la teleenfermería facilita la adherencia al tratamiento y aumenta la capacidad del paciente para cumplir con los esquemas de medicación, debido al autocontrol ^(^ ^16^ ^-^ ^18^ ^,^ ^20^ ^-^ ^21^ ^,^ ^23^ ^-^ ^25^ ^)^ . La telenefrología promete mejorar, aumentar o llenar el vacío en el cuidado renal en diferentes países, en función de los niveles actuales de atención, como lo demuestra un estudio realizado en los Estados Unidos de América, cuya adherencia fue relativamente alta en la muestra, debido a la ventaja del monitoreo electrónico ^(^ ^20^ ^)^ . 

 También se suma la ampliación del conocimiento sobre la ERC ^(^ ^16^ ^-^ ^19^ ^,^ ^21^ ^-^ ^22^ ^,^ ^24^ ^)^ , ya que los efectos de la intervención educativa establecen la efectividad para mejorar la función renal y tratar la enfermedad ^(^ ^22^ ^)^ . El uso del *e-learning* , por ejemplo, es capaz de generar una diferencia significativa en términos de función renal, y se recomienda la utilización del sistema en atención primaria de salud para la educación de los pacientes ^(^ ^22^ ^)^ . 

 Entre las tecnologías asociadas a la telesalud, se destacó el uso de aplicaciones para *smartphones* ^(^ ^17^ ^-^ ^18^ ^,^ ^20^ ^-^ ^21^ ^,^ ^23^ ^,^ ^25^ ^)^ . Estos son capaces de simplificar tareas importantes y apoyar la toma de decisiones del paciente en tiempo real, además de conectarlo con su equipo de atención médica sin ser intrusivos, en ninguna de las partes, por medio de un sistema de alerta dinámico y personalizable ^(^ ^23^ ^)^ , aumentando la adherencia al tratamiento ^(^ ^18^ ^,^ ^20^ ^,^ ^23^ ^-^ ^25^ ^)^ . 

 El uso de sitios *web* ^(^ ^16^ ^,^ ^22^ ^)^ también fue visto como una tecnología asociada a la teleenfermería capaz de promover la autogestión y la adherencia a la medicación ^(^ ^16^ ^)^ , la mejora de la función renal ^(^ ^22^ ^)^ y el tratamiento de la ERC a través de la intervención educativa en el formato *e-learning* ^(^ ^22^ ^)^ , actuando como un repositorio de información sobre la ERC, de fácil acceso, para que el paciente amplíe la comprensión de su enfermedad. 

 Otras estrategias para reducir el número de eventos de hospitalización fueron el uso de atención telefónica ^(^ ^24^ ^)^ y teleconferencias ^(^ ^19^ ^)^ . En el caso de la asistencia telefónica, tanto individual como grupal, se garantiza un espacio dedicado para brindar información, apoyo y la oportunidad a las personas de expresar y compartir sus experiencias de vivir con ERC ^(^ ^29^ ^)^ . Su uso ha logrado un gran éxito a la hora de frenar la progresión de la enfermedad renal diabética y no diabética. La educación multidisciplinaria previa a la diálisis disminuyó la incidencia de diálisis y redujo la mortalidad por todas las causas. Además, después del establecimiento de centros de teleasistencia y prevención de ERC, hubo una reducción drástica en la incidencia de insuficiencia renal terminal en Taiwán ^(^ ^24^ ^)^ . 

 En general, los estudios que tuvieron como objetivo evaluar la adherencia ^(^ ^17^ ^-^ ^18^ ^,^ ^20^ ^,^ ^22^ ^)^ y el autocuidado ^(^ ^16^ ^-^ ^25^ ^)^ mostraron una mejora en los índices analizados, reforzando que las tecnologías de teleenfermería fueron capaces de influir en el conocimiento sobre la ERC ^(^ ^16^ ^-^ ^19^ ^,^ ^21^ ^-^ ^22^ ^,^ ^24^ ^)^ , en la adherencia a la medicación ^(^ ^16^ ^-^ ^18^ ^,^ ^20^ ^-^ ^21^ ^,^ ^23^ ^-^ ^25^ ^)^ , en la dieta ^(^ ^16^ ^-^ ^18^ ^,^ ^21^ ^,^ ^24^ ^-^ ^25^ ^)^ , en el monitoreo de la presión arterial ^(^ ^18^ ^,^ ^23^ ^)^ , en la práctica de ejercicios ^(^ ^16^ ^-^ ^18^ ^,^ ^21^ ^,^ ^24^ ^,^ ^25^ ^)^ y en hábitos de vida, como fumar ^(^ ^18^ ^)^ . Sin embargo, en lo que respecta al conocimiento sobre la ERC, la edad avanzada, el bajo nivel educativo y la duración de la enfermedad influyen en el proceso de aprendizaje del paciente ^(^ ^18^ ^)^ . 

 También cabe resaltar que las tecnologías de fácil uso y alcance son útiles principalmente en pacientes con condiciones asintomáticas ^(^ ^16^ ^)^ , en los cuales la progresión de la enfermedad puede pasar desapercibida por la falta de síntomas perceptibles, y ser negligenciada. Por tanto, este recurso transmite al paciente información y conocimientos necesarios para comprender la ERC, su progresión y gravedad, favoreciendo la adherencia al tratamiento y su papel en la mejora de la calidad de vida ^(^ ^16^ ^,^ ^20^ ^)^ . Asimismo, desde una perspectiva profesional, tener al alcance una herramienta que facilite el seguimiento del paciente permite una intervención más asertiva y rápida por medio de una vigilancia constante, sirviendo de apoyo cuando surgen dudas y brindando contención al paciente ^(^ ^16^ ^)^ . 

 Finalmente, es importante considerar el público objetivo al utilizar nuevas tecnologías ^(^ ^16^ ^)^ , especialmente cuando se trata de la necesidad de conocimientos digitales previos a su uso, como en el caso de aplicaciones, sitios *web* o teleconferencias. 

### El papel de la enfermería en relación con las tecnologías de telesalud

 La teleenfermería no se limita a la teleconsulta, y puede utilizarse para la realización de casos clínicos, gestión de personal, capacitación del equipo y servicio comunitario. Es especialmente útil para enfermeros que no están físicamente presentes, pero cuya orientación es fundamental para brindar cuidados seguros, garantizando privacidad y confidencialidad en un ambiente cómodo ^(^ ^30^ ^)^ . 

 Entre los resultados de la revisión, el enfermero demostró ser una parte central de la práctica de telesalud. En estudios en los que la teleenfermería fue el foco ^(^ ^16^ ^-^ ^17^ ^,^ ^25^ ^)^ , su papel como líder en la gestión de la tecnología fue fundamental para su implementación y efectividad en el cuidado del paciente con ERC. Incluso en el equipo multidisciplinario, el enfermero mantiene la relevancia de su rol en telesalud, brindando apoyo, orientación, educación y seguimiento del estado de salud ^(^ ^18^ ^,^ ^23^ ^-^ ^24^ ^)^ . 

 El uso de la teleenfermería facilita la implementación de pautas y capacitación entre los cuidadores ^(^ ^27^ ^)^ y proporciona educación y conciencia al paciente sobre la participación activa en su propio cuidado, ofreciendo mejores prácticas para involucrarse en el autocuidado (autogestión) ^(^ ^22^ ^,^ ^25^ ^)^ . Esto se revela como una práctica de investigación, atención y gestión, que puede promover la adherencia, la mejora del acceso al cuidado y la seguridad del paciente, así como la creación de una red de comunicación e información interprofesional, incentivando esta nueva práctica y la producción e innovación tecnológica ^(^ ^28^ ^)^ . 

 Por tanto, es beneficioso para el enfermero conocer a su paciente y comprender que, a pesar de todos los cambios que trae la enfermedad, hay vida más allá de la ERC. El enfermero también necesita cuidar sus propios valores y actitudes a la hora de crear una relación mutua con el individuo ^(^ ^29^ ^)^ . 

 Se destaca que las implicaciones de sensibilizar sobre el involucramiento del paciente, apoyando prácticas de cuidado que promuevan su compromiso y empoderamiento, además de aportar a la reflexión de la actitud del paciente renal hacia la vigilancia y control de su propio tratamiento, fomentan el cuidado en salud, como resultado de la colaboración entre profesionales y usuarios, para contribuir a romper el estatus de “paciente pasivo”, subyacente al modelo de cuidado centrado en la enfermedad, tan presente en los servicios de salud ^(^ ^31^ ^)^ . 

 En resumen, dado el potencial de la atención a distancia, el trabajo remoto se presenta como una innovación en la práctica de enfermería y en el área de la salud, al mismo tiempo que un desafío para los profesionales ^(^ ^32^ ^)^ , que brinda oportunidades para una mejor organización del servicio de salud en una situación de crisis, como ocurrió durante la pandemia de COVID-19, permitiendo a los involucrados en el cuidado del paciente atenderlo de forma segura y con calidad. 

 Considerando que la teleenfermería aún es una práctica en desarrollo en varios países ^(^ ^33^ ^)^ , y que la mayoría de los estudios no tuvieron un marco temporal longitudinal, es posible observar como un vacío de conocimiento la falta de resultados a largo plazo de la implementación de la teleconsulta en la regresión o estabilización de la ERC, en particular si consideramos el uso de innovaciones tecnológicas, principalmente en las primeras etapas de la enfermedad. 

## Conclusión

Se evidenció que el uso de la telesalud por parte de la enfermería es relevante en el contexto de la salud, tanto en términos sociales como económicos, especialmente considerando la gravedad de la ERC como condición de salud global. Su uso es capaz de reducir la progresión temprana de la pérdida de función renal, pero aún está poco explorado en la literatura nacional e internacional, lo que representa un vacío de conocimiento a llenar en futuras investigaciones. Los estudios han demostrado que su implementación ayuda y apoya a los profesionales de la salud, brindando inferencias y orientaciones sobre una asistencia rápida, segura y efectiva, incluso de forma remota, a través de plataformas, teleconferencias, telemensajería y portales de servicios de atención de tecnología en salud.

Actuando como un medio de prevención y promoción de la salud a distancia, la teleenfermería se presenta como una estrategia capaz de promover la adherencia al tratamiento y al autocuidado en pacientes con insuficiencia renal crónica sometidos a tratamiento conservador. Favorece la mejora de la práctica clínica y del conocimiento, y el desarrollo de tecnologías e innovación en salud en el campo de la enfermería, contribuyendo al avance científico y al desarrollo de políticas públicas en el área de enfermedades crónicas no transmisibles, y por tanto puede generar un impacto positivo en la salud pública.

Finalmente, dadas las brechas identificadas, se recomienda que en el futuro se realicen estudios para monitorear el uso de tecnologías en salud para mejorar el autocuidado y la adherencia al tratamiento de pacientes con ERC, desde sus etapas iniciales de prediálisis hasta el ingreso a la terapia de reemplazo renal.

# Translation: Uso da Telenfermagem no tratamento conservador de pacientes com insuficiência renal crônica: revisão de escopo ^*^

**DOI:** 10.1590/1518-8345.7013.4360

**Authors:** Aline de Oliveira Biancamano, Alessandra Conceição Leite Funchal Camacho, Elaine Antunes Cortez, Harlon França de Menezes, Yasmin Saba de Almeida, Cristiele Costa da Matta Rocha

**Affiliations:** ^1^ Universidade Federal Fluminense, Escola de Enfermagem Aurora de Afonso Costa, Niterói, RJ, Brasil.; ^2^ Ministério da Saúde, Hospital Federal dos Servidores do Estado, Serviço de Nefrologia, Rio de Janeiro, RJ, Brasil.; ^3^ Universidade Federal Fluminense, Escola de Enfermagem Aurora de Afonso Costa, Departamento de Fundamentos de Enfermagem e Administração, Niterói, RJ, Brasil.; ^4^ Universidade Federal Fluminense, Escola de Enfermagem Aurora de Afonso Costa, Departamento de Enfermagem Materno-Infantil e Psiquiátrica, Niterói, RJ, Brasil.

**Keywords:** Telenfermagem, Cooperação e Adesão ao Tratamento, Autocuidado, Insuficiência Renal Crônica, Tratamento Conservador, Tecnologia Biomédica

## Abstract

**Objetivo:**

mapear as evidências do uso da telenfermagem na adesão ao tratamento e promoção do autocuidado em pacientes com insuficiência renal crônica em tratamento conservador.

**Método:**

estudo de revisão de escopo, com seleção de documentos indexados em oito bases de dados e dois catálogos de teses e dissertações. Adicionalmente, foram consultadas as listas de referências dos estudos selecionados. A seleção e a análise dos estudos foram realizadas no software Rayyan por meio de dupla investigação, independente e cega.

**Resultados:**

foram encontradas 56 publicações elegíveis e foram selecionadas dez para compor o estudo. Destacaram-se entre as intervenções de telenfermagem: aplicativos para celular, websites, plataforma digital, suporte telefônico e teleconferência.

**Conclusão:**

evidenciou-se que o uso da Telessaúde pela Enfermagem é relevante para o âmbito da saúde, porém ainda pouco explorado pela literatura nacional e internacional, representando uma lacuna no conhecimento a ser preenchida em futuras pesquisas. Os estudos demonstraram que sua implementação auxilia e dá suporte aos profissionais da saúde, proporcionando inferências e orientações para uma assistência rápida, segura e efetiva, mesmo que à distância. A telenfermagem se apresenta como estratégia capaz de promover a adesão ao tratamento e ao autocuidado em pacientes com insuficiência renal crônica em tratamento conservador.

## Destaques

**(1)** Desenvolve estratégias de inovação em saúde no tratamento à distância.

**(2)** Oferece suporte aos profissionais com medidas preventivas, minimizando custos.

**(3)** Estimula a prática de autogerenciamento do cuidado.

**(4)** Proporciona inferências e orientações para uma assistência rápida e segura.

**(5)** Contribui para o avanço de políticas públicas na área de doenças não-transmissíveis.

## Introdução

 A insuficiência renal crônica é definida pela presença de alterações da estrutura ou das funções dos rins, com ou sem alteração da filtração glomerular, por um período maior que três meses ^(^ ^1^ ^)^ . É a taxa de filtração glomerular (TFG) que tem maior correlação com os desfechos clínicos, apesar da creatinina ser o melhor marcador da função renal ^(^ ^2^ ^)^ . O nível da TFG varia conforme a idade, o sexo e a massa muscular. Com o avançar da idade, é possível observar uma diminuição da TFG, podendo essa redução se manifestar bem antes do início dos sintomas e estar associada à gravidade da doença renal crônica (DRC) ^(^ ^3^ ^)^ . 

 A DRC é progressiva e irreversível até o momento, porém pode ser tratada inicialmente por meio de medidas terapêuticas conservadoras, que consistem em medidas clínicas utilizadas para retardar a piora da função renal, reduzir os sintomas e prevenir suas complicações ^(^ ^4^ ^)^ . Iniciar o tratamento conservador de forma precoce aumenta as chances de preservar a função renal por um período de tempo mais prolongado ^(^ ^5^ ^)^ . 

 O tratamento conservador aborda a gestão dos fatores de risco visando prevenir a progressão da doença renal em seus estágios iniciais, que variam do 1 ao 3, quando a TFG se situa entre ≥90 e 30 mL/min/1,73 m ^2^ . Ele também engloba a fase pré-diálise nos estágios 4 e 5, não dialíticos, quando a TFG varia de 29 a <15 mL/min/1,73 m ^2^ , e inclui a terapia renal substitutiva ao alcançar o estágio 5 dialítico, no qual a TFG já está abaixo de 15 mL/min/1,73 m ^2^^(^ ^2^ ^)^ . 

 O controle da DRC pelo paciente está diretamente relacionado às mudanças nos hábitos de vida, e estas são efetivamente alcançadas quando inseridas nas atividades de cuidado do dia a dia que a pessoa realiza para melhorar a própria saúde (autocuidado) ^(^ ^6^ ^)^ . 

 Como os estágios iniciais da DRC são tipicamente assintomáticos e facilmente ignorados, a conscientização com relação à doença é baixa, e os pacientes geralmente atrasam o tratamento e o negligenciam. Desta forma, os profissionais de saúde que cuidam de usuários em condições crônicas precisam de novos modelos que os auxiliem a desenvolver competências avançadas de comunicação ^(^ ^3^ ^)^ . 

 Com o advento da pandemia e a necessidade de manter o isolamento e o distanciamento social, o Conselho Federal de Enfermagem, em 2020 ^(^ ^7^ ^)^ , decidiu normatizar a prática da teleconsulta de enfermagem no Brasil e, posteriormente, em 2022, criou a Resolução nº 696/2022 ^(^ ^8^ ^)^ , normatizando a telenfermagem com a atuação do enfermeiro na saúde digital. A telenfermagem integra a telessaúde e é caracterizada pelo uso de recursos tecnológicos para a realização da prática de enfermagem à distância, nas dimensões: assistencial, educacional ou de pesquisa ^(^ ^9^ ^)^ . 

 Nessa perspectiva, uma revisão integrativa ^(^ ^10^ ^)^ avaliou o uso da telenfermagem na assistência a pacientes crônicos e evidenciou que esta tecnologia proporcionou benefícios aos pacientes, aos profissionais e ao sistema de saúde. No que tange aos pacientes, observou-se sensação de empoderamento, melhora do gerenciamento da doença, diminuição da ansiedade, melhora da qualidade de vida e aumento da adesão medicamentosa. No que se refere aos profissionais, evidenciou-se aumento da comunicação e apoio, melhor manejo dos sintomas, possibilitando diagnóstico e intervenção precoces, e economia do tempo de trabalho. Por último, para o sistema de saúde, detectou-se a redução da taxa de internações hospitalares e consultas ambulatoriais. 

 Entendendo as potencialidades da telenfermagem para o cuidado de pacientes com DRC e a urgência do início do tratamento conservador, estudos que procuram retratar seu uso se tornam essenciais para a saúde pública. Contudo, a busca por aprofundamento da temática ainda é desafiadora, uma vez que há uma escassez de materiais devido ao ineditismo da prática, e a pouca literatura disponível se encontra dispersa ou de difícil acesso. Assim, esta revisão de escopo se fundamenta na iminência de trazer visibilidade e compreensão ao conceito central do estudo, amparada pela lacuna de conhecimento identificada por meio de buscas prévias no *Prospero* , *Cochrane Database of Systematic Reviews* e *JBI Evidence Synthesis* , cujos resultados demonstram não haver revisões, concluídas ou em andamento, com esse enfoque. 

Sendo assim, justifica-se a realização desta revisão de escopo, que tem por objetivo mapear as evidências do uso da telenfermagem na adesão ao tratamento e promoção do autocuidado em pacientes com insuficiência renal crônica em tratamento conservador. Este estudo pode contribuir para a difusão de uma nova abordagem clínico-prática para a promoção da saúde no tratamento conservador em pacientes com insuficiência renal crônica.

## Método

### Delineamento do estudo

 Trata-se de um estudo de revisão de escopo, conforme o método de revisão proposto pela colaboração JBI ^(^ ^11^ ^)^ . Este método permite fornecer uma síntese abrangente e imparcial de estudos relevantes dentro dos limites de um único documento, usando métodos rigorosos e transparentes ^(^ ^12^ ^)^ . Para o relato da revisão, utilizou-se o *Preferred Reporting Items for Systematic reviews and Meta-Analyses extension for Scoping Reviews* (Prisma-ScR) *checklist* ^(^ ^13^ ^)^ . 

Assim, esta investigação tem como objetivo principal mapear as evidências de uso da telenfermagem, norteada pela seguinte questão: Como o uso da telenfermagem influencia a adesão ao tratamento e a promoção do autocuidado em pacientes com insuficiência renal crônica em tratamento conservador? Para a construção da pergunta de pesquisa, utilizou-se a estratégia mnemônica população, conceito e contexto (PCC). Foram definidos: população - pessoas com insuficiência renal crônica em tratamento conservador; conceito - telenfermagem; e contexto - adesão ao tratamento e promoção do autocuidado.

### Protocolo e registro

 Inicialmente, realizou-se um protocolo de pesquisa com o objetivo de garantir o rigor metodológico da revisão, e seus componentes compuseram as seguintes fases: definição do objetivo e pergunta de pesquisa; definição dos critérios de elegibilidade (inclusão e exclusão); planejamento da estratégia de busca e seleção dos estudos com relação aos descritores específicos de cada base de dados; identificação dos estudos e seleção dos estudos; extração dos dados; mapeamento dos dados; e sumarização dos resultados. O protocolo final foi registrado na plataforma *Open Science Framework* (OSF) ^(^ ^14^ ^)^ . 

### Critérios de elegibilidade

De acordo com o acrônimo PCC, os seguintes critérios de elegibilidade foram estabelecidos: inclusão - estudos primários de corte transversal, observacionais, ensaios clínicos, estudos quase-experimentais e randomizados, realizados com pacientes adultos (18 anos ou mais), publicados em qualquer idioma, sem delimitação de período; exclusão - cartas ao editor e editoriais; estudos que abordassem apenas insuficiência renal crônica em tratamento conservador, mas não abordassem a telenfermagem; estudos que abordassem terapia dialítica sem tratamento conservador como um dos métodos ou método exclusivo; estudos que não tivessem pelo menos um profissional enfermeiro atuando ou desenvolvendo a tecnologia associada à telessaúde; e, ainda, que a abordagem não se tratasse de algum dispositivo de telessaúde.

Cabe destacar que, embora estudos secundários tenham sido considerados inicialmente para inclusão na revisão, nenhuma publicação com esse delineamento respondeu à questão de pesquisa.

### Fontes de informação e pesquisa

 A busca foi realizada em 6 de janeiro de 2023 e atualizada em 23 de abril de 2024. Primeiramente, foram consultadas as seguintes bases de dados eletrônicas: Base de Dados em Enfermagem (BdEnf), *Índice Bibliográfico Español en Ciencias de la Salud* (Ibecs) e Literatura Latino-americana e do Caribe em Ciências da Saúde (LILACS), por meio da Biblioteca Virtual em Saúde (BVS); *Cumulative Index to Nursing and Allied Health Literature* (CINAHL), Embase, PubMed, *Scopus* e *Web of Science Core Collection* ( *WoS Core Collection* ). Posteriormente, foram consultados dois catálogos de dissertações e teses para levantamento da literatura cinzenta: um internacional, a *Networked Digital Library of Theses and Dissertations* (NDLTD), e outro nacional, a Biblioteca Digital Brasileira de Teses e Dissertações (BDTD). Adicionalmente, foram consultadas as listas de referências dos estudos selecionados. 

 Para a estratégia de busca, utilizaram-se descritores combinados com os operadores booleanos “AND” e/ou “OR”, conforme necessidade em cada base. Para a seleção dos termos no idioma inglês, foi consultado o *Medical Subject Headings* (MeSH), e nos idiomas português, francês e espanhol, os Descritores em Ciências da Saúde (DeCS). Desta forma, compondo a estratégia de busca, estiveram os seguintes termos: telenfermagem; consulta remota; cooperação do paciente; autocuidado; e insuficiência renal crônica ( Figura 1 ). O descritor “tratamento conservador” não foi adicionado à estratégia de busca, uma vez que seus resultados captam estudos que não são voltados à sua definição de prevenção. 

**Figura 1:** - Estratégia de busca implementada na base de dados PubMed. Niterói, RJ, Brasil, 2023

**Bases de dados**	**Estratégia**
PubMed	((“Renal Insufficiency, Chronic”[mh] OR Chronic Kidney Disease ^*^ [tiab] OR Chronic Kidney Insufficiency ^*^ [tiab] OR Chronic Renal Disease ^*^ [tiab] OR Chronic Renal Insufficiency ^*^ [tiab]) AND ((“Remote Consultation”[mh] OR Remote Consultation[tiab] OR “Asynchronous Teleconsultation”[tiab] OR Teleconsultation[tiab] OR Telemonitoring[tiab] OR “Remote Monitoring”[tiab] OR “Remote Patient Monitoring”[tiab] OR Tele-Monitoring[tiab] OR Telehealth Monitoring[tiab] OR Telemedicine Monitoring[tiab] OR “Telemedicine”[mh] OR Telemedicine[tiab] OR “Mobile Health”[tiab] OR mHealth[tiab] OR Telehealth[tiab] OR eHealth[tiab] OR “Pervasive Health”[tiab] OR Telecare[tiab] OR Tele-Service ^*^ [tiab] OR Teleservices ^*^ [tiab] OR “Connected Health”[tiab] OR “Digital Health”[tiab] OR “telemedicine”[tiab] OR “virtual medicine”[tiab] OR “distant monitoring”[tiab] OR “distant patient monitoring”[tiab] OR “remote monitoring”[tiab] OR “remote patient monitoring”[tiab] OR “remote patient surveillance”[tiab] OR “tele monitoring”[tiab] OR “tele surveillance”[tiab] OR telesurveillance[tiab] OR “Telenursing”[mh] OR Telenursing[tiab] OR “Tele nursing”[tiab] OR tele-nursing[tiab] OR “virtual nursing”[tiab]))) AND ((“Self Care”[mh] OR “Self Care”[tiab] OR Self-Care[tiab] OR “Patient Compliance”[mh] OR “Patient Compliance”[tiab] OR Client Adherence ^*^ [tiab] OR Client Compliance ^*^ [tiab] OR Adherent Patient ^*^ [tiab] OR Patient Adherence ^*^ [tiab] OR Patient Cooperation[tiab] OR Patient Nonadherence[tiab] OR Patient Noncompliance[tiab] OR Therapeutic Compliance ^*^ [tiab] OR Treatment Compliance ^*^ [tiab] OR “Treatment Adherence and Compliance”[tiab] OR “Therapeutic Adherence”[tiab] OR “Treatment Adherence”[tiab] OR “Medication Adherence”[mh] OR Drug Adherence [tiab] OR Drug Compliance[tiab] OR Medication Compliance[tiab] OR Medication Adherence[tiab] OR Medication Nonadherence[tiab] OR Medication Noncompliance[tiab] OR Medication Persistence[tiab]))

Destaca-se que na BVS e na BDTD foi necessário dividir a estratégia de busca em duas partes: uma com os descritores em português, espanhol e francês, e outra com os descritores apenas em inglês. Isso ocorreu, pois, ao lidar com estratégias de busca quadrilíngues, o limite de caracteres buscáveis foi atingido.

### Seleção das fontes de evidência

 Após a busca nas bases e nas fontes, procedeu-se à seleção dos documentos, norteada pela questão da pesquisa. Os resultados obtidos nas fontes de informação foram exportados para o programa *EndNote* versão *desktop* para remoção dos documentos duplicados. Posteriormente, os mesmos foram adicionados ao *software Rayyan* ^(^ ^15^ ^)^ , desenvolvido pelo *Qatar Computing Research Institute* , possibilitando a seleção e triagem dos estudos, conforme critérios pré-definidos. Também foram consideradas as recomendações do Prisma-ScR *checklist* ^(^ ^13^ ^)^ . 

 A seleção dos estudos foi realizada no *software Rayyan* ^(^ ^15^ ^)^ , por meio de dupla investigação (por pares), de forma independente e às cegas. Deste modo, primeiro foram avaliados títulos e resumos, observando se atendiam aos critérios de inclusão e exclusão previamente definidos no protocolo. Aqueles que cumpriam os requisitos foram lidos na íntegra, e uma nova reunião de consenso foi realizada para a resolução de conflitos. 

### Tratamento, análise e extração de dados

Após a definição da amostra, os estudos selecionados tiveram seu conteúdo lido minuciosamente, na íntegra, por repetidas vezes, de forma a permitir a extração e o mapeamento dos dados.

 A extração de dados foi realizada por meio de um roteiro elaborado pelos autores, dando origem a um banco de dados constituído pela caracterização das publicações e informações relevantes ao objeto de pesquisa, contendo: autoria/citação, ano de publicação, tipo de publicação, idioma, país, objetivo do estudo, população do estudo, delineamento metodológico e principais desfechos. Para garantir a adequação aos critérios de elegibilidade, o roteiro também incluiu elementos correlatos ao acrônimo PCC, como: estágio da TFG (P), intervenções e tecnologias utilizadas associadas à telenfermagem (C), e adesão (ou não adesão) ao tratamento e promoção do autocuidado nos resultados dos estudos (C). O banco de dados foi confeccionado no programa *Microsoft Excel* , e teve seu conteúdo preenchido por um dos pesquisadores e revisado por seu par. 

### Síntese dos resultados

Nesta pesquisa, os dados coletados foram submetidos a um resumo narrativo e análise estatística descritiva. Para apresentar uma visão clara e concisa dos achados, os resultados foram organizados em tabelas e figuras. Essa abordagem permitiu uma compreensão aprofundada dos padrões e tendências presentes nas informações dos estudos, possibilitando tanto mapeá-las quanto consolidar as conclusões deste estudo.

### Aspectos éticos

Com relação aos aspectos éticos da pesquisa, não houve discriminação na seleção dos artigos ou estudos, respeitando-se o critério de cegamento individual.

## Resultados

 A pesquisa identificou nas fontes de informação um total de 1.046 estudos potencialmente elegíveis. Estes foram exportados para o programa *EndNote* versão *desktop* , no qual foi possível remover 154 estudos duplicados. Ao final da triagem no *software Rayyan* ^(^ ^15^ ^)^ , foram selecionados para compor a revisão de escopo um total de nove artigos ^(^ ^16^ ^-^ ^24^ ^)^ e uma dissertação ^(^ ^25^ ^)^ , desenvolvidos em sete países diferentes, entre os anos de 2011 a 2023. Cabe destacar que, apesar das listas de referências terem sido consultadas, não foi possível captar estudos que respondessem aos critérios de elegibilidade. O processo de busca e seleção dos estudos foi descrito no fluxograma Prisma-ScR ^(^ ^13^ ^)^ ( Figura 2 ). 

 Conforme a Tabela 1 , no que se refere às características dos dez estudos selecionados, o primeiro foi publicado em 2011 e posteriormente em 2015, e os demais foram publicados de forma descontínua até 2023. A maior produção ocorreu em 2019 e 2023, ambos com dois artigos. Todos os estudos são quantitativos, sendo a maioria de coorte ou ensaios randomizados controlados. Quanto ao idioma de publicação, nove encontravam-se publicados em língua inglesa e um em chinês. 

 Três estudos tiveram o enfermeiro como foco da intervenção ^(^ ^16^ ^-^ ^17^ ^,^ ^25^ ^)^ . Quatro tiveram suas tecnologias desenvolvidas por um ou mais enfermeiros ^(^ ^19^ ^-^ ^22^ ^)^ . Já nos demais estudos, o enfermeiro fez parte da equipe multiprofissional, porém mantendo sua relevância no processo de telessaúde ^(^ ^18^ ^,^ ^23^ ^-^ ^24^ ^)^ . No que tange às formas nas quais a telessaúde foi empregada, estiveram presentes: aplicativos para celular (60%), *websites* (20%), suporte telefônico (10%) e teleconferência (10%), como pode ser observado na figura abaixo ( Figura 3 ). 

**Figura 2:** - Fluxograma PRISMA-ScR ^(^ ^13^ ^)^ . Niterói, RJ, Brasil, 2024

**Tabela 1:** - Distribuição dos estudos incluídos conforme país, tipo de estudo, abordagem e ano de publicação. Niterói, RJ, Brasil, 2023

**Características**	**N** ^*^	**%** ^†^
**País** Canadá China Estados Unidos da América Irã Irlanda Reino Unido Taiwan	1 1 1 1 1 2 3	10 10 10 10 10 20 30
**Tipo de estudo** Ensaios randomizados controlados Ensaio clínico controlado não randomizado Pesquisas de desenvolvimento tecnológico Estudo de coorte Estudo longitudinal de viabilidade Estudo de intervenção pré-pós teste Estudo observacional	2 1 1 3 1 1 1	20 10 10 30 1 1 1
**Abordagem** Quantitativa	10	100
**Ano de publicação** 2011 2015 2016 2017 2019 2020 2021 2023	1 1 1 1 2 1 1 2	10 10 10 10 20 10 10 20
**Estágio** ^‡^ **da TFG** ^§^ Estágio 1 Estágio 2 Estágio 3 Estágio 4 (não dialítico) Estágio 5 (não dialítico) Não contabilizado	1 0 5 1 2 1	10 0 50 10 20 10
**Adesão ao tratamento** Sim Não Não contabilizado	4 0 6	40 0 60
**Autocuidado** ^||^ Autogestão do medicamento Autogestão de problemas metabólicos Conhecimento da DRC ^¶^ Dieta Exercício Hábito de fumar Monitoramento da PA ^**^	8 1 7 6 6 1 2	80 10 70 60 60 10 20

^*^ n = Frequência absoluta;

^†^ Frequência relativa;

^‡^ Estadiamento calculado a partir da média da TFG, quando não apresentado pelo estudo. Quando presente, considerou-se o estágio mais prevalente;

^§^ TFG = Taxa de filtração glomerular;

^||^ Houve indicação de um ou mais itens por estudo;

^¶^ DRC = Doença renal crônica;

^**^ PA = Pressão arterial

**Figura 3:** - Destaques das principais intervenções. Niterói, RJ, Brasil, 2023

**Autoria**	**Intervenções**	**Tipo de intervenção**
Reston, et al. (2023) ^(^ ^16^ ^)^	*Website CareKnowDo* . Site com 3 módulos distintos: (1) *Mind Matters* : projetado para lidar com o mau humor; (2) Questões de estilo de vida: abordando principalmente dieta, exercício e como estes afetam a DRC ^*^ ; e (3) Assuntos de Medicação: abrangendo a adesão à medicação anti-hipertensiva. Cada módulo incluiu atividades e ferramentas baseadas na internet, com base na terapia cognitivo-comportamental e em outras técnicas de mudança de comportamento baseadas em evidências. Eles também continham conteúdo psicoeducacional desenvolvido para educar os pacientes sobre a DRC ^*^ e abordar as principais crenças inúteis que impactam o comportamento de autogestão. Os pacientes também receberam uma linha de enfermagem (telenfermagem) para dúvidas ou preocupações.	*Website*
Liu, et al. (2023) ^(^ ^17^ ^)^	Sistema inteligente *KidneyOnline* , desenvolvido para *smartphones* . Serviços prestados: (1) Interpretação da condição da doença e orientação correspondente; (2) *Check-ups* regulares; (3) Avisos antecipados; (4) Campos de perguntas e respostas em tempo real potencializados por gráficos de conhecimento; e (5) Lembretes clínicos. Trata-se de um sistema de gerenciamento colaborativo liderado por enfermeiros e orientado ao paciente, como um complemento às visitas clínicas regulares para pacientes com DRC ^*^ .	Aplicativo móvel
Tsai, et al. (2021) ^(^ ^18^ ^)^	Aplicativo móvel chamado iCKD, que possui vários recursos principais, incluindo testes fisiológicos domiciliares, monitoramento de sinais, educação em saúde sobre doenças e autocuidado, análise nutricional, lembrete de medicação e alarmes, e um sistema de alerta. O aplicativo permite que a equipe multiprofissional (incluindo o enfermeiro) analise e monitore remotamente o estado de saúde de um paciente com dados autorregistrados, possibilitando o envio de *feedbacks* atempados com lembretes *online* .	Aplicativo móvel
Winocour, et al. (2020) ^(^ ^19^ ^)^	Sessão educacional de telessaúde via *Skype* , com duração de duas horas, que teve como intuito revisar casos virtuais de telessaúde. Para tanto, foram pré-selecionados um total de 20 casos clínicos para discussão.	Teleconferência
Ellis, et al. (2019) ^(^ ^20^ ^)^	Sistema *mHealth* : dispositivo de botão inteligente que auto-monitora a tomada de medicamentos, aplicativo de *smartphone* complementar, algoritmo de computador usado para determinar a adesão e, em seguida, enviar um suporte de autogestão ( *self-management support* ) padrão ou personalizado por mensagem de texto (serviço de mensagens curtas) com base no horário de uso do medicamento. Mensagens de texto *self-management support* padrão indicavam que o aplicativo para *smartphone* registrou o pressionamento do botão, enquanto as mensagens de texto *self-management support* personalizadas incentivavam a formação de hábitos e o pensamento sistêmico com base no horário em que os medicamentos foram tomados.	Aplicativo móvel
Doyle, et al. (2019) ^(^ ^21^ ^)^	O aplicativo *MiKidney* registra detalhes pessoais, histórico médico, sangue, peso e lista de medicamentos atuais. Ele fornece informações sobre DRC ^*^ , medicação para DRC ^*^ , dieta renal, opções de tratamento de substituição renal, gerenciamento de sintomas, e manutenção da saúde. O aplicativo inclui um rastreador de exercícios e um registro diário dos exercícios realizados, alertas de lembretes e uma seção de notas para registrar quaisquer dúvidas ou questões a serem discutidas com a equipe disciplinar. Além disso, o aplicativo possui um esquema de pontuação “ *My Renal Rating* ”, com um sistema de semáforos que fornece *feedback* aos usuários, incluindo mensagens motivacionais.	Aplicativo móvel
Barahimi, et al. (2017) ^(^ ^22^ ^)^	*E-learning* como intervenção educacional na melhora da função renal e no tratamento da DRC ^*^ . O modelo de *e-learning* utilizado no estudo foi o modelo ADDIE ^†^ , acrônimo para análise, design, desenvolvimento, implementação e *evaluation* (avaliação). O conteúdo relacionado à DRC ^*^ (definição, diagnóstico, estadiamento, fatores de risco, complicações, cuidados e acompanhamento) foi identificado por meio das *guidelines* do “ *Kidney Disease: Improving Global Outcomes* ” ^(^ ^26^ ^)^ .	*Website*
Ong, et al. (2016) ^(^ ^23^ ^)^	Sistema de autogerenciamento baseado em aplicativo de *smartphone* , projetado e desenvolvido como complemento aos cuidados habituais de DRC ^*^ . O aplicativo para *smartphone* tinha como alvo quatro elementos comportamentais: monitoramento da PA ^‡^ , gerenciamento de medicamentos, avaliação de sintomas, e acompanhamento de resultados laboratoriais. As mensagens de e-mail eram enviadas automaticamente quando as respostas exigiam ações mais urgentes, e os destinatários eram determinados pela gravidade médica (por exemplo, apenas enfermeiro e/ou farmacêutico ou enfermeiro, farmacêutico e médico).	Aplicativo móvel
Chen, et al. (2015) ^(^ ^24^ ^)^	O suporte de autogestão compreendia informações de saúde, educação do paciente, suporte telefônico, e auxílio de um grupo de apoio. O suporte telefônico envolveu uma ligação telefônica semanal para melhorar o autogerenciamento da DRC ^*^ e garantir o acompanhamento oportuno. Os enfermeiros ficaram responsáveis pelas palestras de educação em saúde, pelos grupos de apoio e pelo suporte telefônico.	Suporte telefônico
Lu (2011) ^(^ ^25^ ^)^	Sistema móvel de gerenciamento de saúde e autocuidado da DRC ^*^ , desenvolvido para *smartphones* para auxiliar os pacientes com o automonitoramento da doença. A equipe de enfermagem pode monitorar o estado de saúde dos pacientes, enviar *feedbacks* e sugestões via mensagem instantânea através dos *smartphones* , bem como pesquisar registros e fazer *upload* de relatórios de inspeção pela *web* .	Aplicativo móvel

^*^ DRC = Doença renal crônica;

^†^ ADDIE = Acrônimo para análise, *design* , desenvolvimento, implementação e *evaluation* (avaliação);

^‡^ PA = Pressão arterial

 Os estudos levantados nesta revisão revelaram que intervenções tecnológicas contribuíram para a melhora da saúde do paciente com DRC, por meio: do aperfeiçoamento no monitoramento da doença ^(^ ^16^ ^-^ ^18^ ^,^ ^20^ ^-^ ^21^ ^,^ ^23^ ^,^ ^25^ ^)^ ; do retardo da progressão renal ^(^ ^21^ ^,^ ^24^ ^)^ e redução dos eventos de hospitalização em estágio avançado ^(^ ^24^ ^)^ ; de que o cuidado multidisciplinar coordenado ofereceu um gerenciamento ideal do paciente ^(^ ^18^ ^,^ ^24^ ^-^ ^25^ ^)^ e melhorou a sobrevida renal ^(^ ^24^ ^)^ ; do alto nível de aceitação e satisfação pelos pacientes ^(^ ^23^ ^)^ ; da sinalização de atendimento prioritário para os pacientes que precisam de mais atenção ^(^ ^23^ ^)^ ; da melhora da função renal ^(^ ^19^ ^,^ ^21^ ^-^ ^22^ ^,^ ^24^ ^)^ ; de um melhor controle da pressão arterial ^(^ ^16^ ^-^ ^17^ ^,^ ^19^ ^)^ e glicemia ^(^ ^16^ ^,^ ^19^ ^)^ , redução de peso ^(^ ^19^ ^,^ ^21^ ^-^ ^22^ ^)^ e resultado cardiorrenal ^(^ ^19^ ^)^ ; e da diminuição de custos do tratamento, evitando o deslocamento para o hospital ou unidade de saúde ^(^ ^17^ ^,^ ^23^ ^)^ . 

## Discussão

### O uso das tecnologias de telenfermagem enquanto ferramentas de adesão e autocuidado no tratamento conservador da DRC

 As consequências da DRC continuam a representar enormes desafios em todo o mundo. Acesso e qualidade de cuidados para DRC permanecem abaixo do ideal em todas as configurações, em parte devido ao acesso limitado a especialistas apropriados para prestar cuidados ^(^ ^27^ ^)^ . Ao enfrentar esses desafios, a telenfermagem pode prolongar a vida útil das pessoas, por meio da otimização das operações, compartilhamento da informação interprofissional, detecção precoce de problemas de saúde e tratamento ^(^ ^28^ ^)^ . 

 Os resultados evidenciam que o uso da telenfermagem facilita a adesão ao tratamento e aumenta a capacidade do paciente de cumprir os regimes medicamentosos, por conta do automonitoramento ^(^ ^16^ ^-^ ^18^ ^,^ ^20^ ^-^ ^21^ ^,^ ^23^ ^-^ ^25^ ^)^ . A telenefrologia promete melhorar, aumentar ou preencher a lacuna no cuidado renal em diferentes países, com base nos níveis atuais de atendimento, como demonstrou um estudo desenvolvido nos Estados Unidos da América, cuja adesão foi relativamente alta na amostra devido à vantagem do monitoramento eletrônico ^(^ ^20^ ^)^ . 

 Acrescenta-se também a ampliação do conhecimento sobre a DRC ^(^ ^16^ ^-^ ^19^ ^,^ ^21^ ^-^ ^22^ ^,^ ^24^ ^)^ , uma vez que os efeitos da intervenção educacional estabelecem a eficácia para a melhora da função renal e do tratamento da doença ^(^ ^22^ ^)^ . O uso do *e-learning,* por exemplo, é capaz de gerar uma diferença significativa em termos de função renal, sendo recomendada a utilização do sistema na atenção primária à saúde para a educação dos pacientes ^(^ ^22^ ^)^ . 

 Dentre as tecnologias associadas à telessaúde, o uso de aplicativos para *smartphones* se destacou ^(^ ^17^ ^-^ ^18^ ^,^ ^20^ ^-^ ^21^ ^,^ ^23^ ^,^ ^25^ ^)^ . Estes são capazes de simplificar tarefas importantes e apoiar a tomada de decisão do paciente em tempo real, além de conectá-lo com sua equipe de saúde sem serem intrusivos, em nenhum dos lados, por meio de um sistema de alerta dinâmico e personalizável ^(^ ^23^ ^)^ , aumentando a adesão ao tratamento ^(^ ^18^ ^,^ ^20^ ^,^ ^23^ ^-^ ^25^ ^)^ . 

 O uso de *websites* ^(^ ^16^ ^,^ ^22^ ^)^ também foi visto como tecnologia associada à telenfermagem capaz de promover a autogestão e adesão à medicação ^(^ ^16^ ^)^ , a melhora da função renal ^(^ ^22^ ^)^ e o tratamento da DRC através da intervenção educativa no formato *e-learning* ^(^ ^22^ ^)^ , agindo como um repositório de informações à respeito da DRC, de fácil acesso, para que o paciente amplie a compreensão sobre a sua doença. 

 Outras estratégias para a redução do número de eventos de hospitalizações foram o uso de suporte telefônico ^(^ ^24^ ^)^ e teleconferências ^(^ ^19^ ^)^ . No caso do atendimento telefônico, tanto individual quanto em grupo, garante-se um espaço dedicado para fornecer informações, suporte e a oportunidade para que os indivíduos expressem e compartilhem suas experiências de viver com DRC ^(^ ^29^ ^)^ . Seu uso alcançou grande sucesso em retardar a progressão da doença renal diabética e não diabética. A educação pré-diálise multidisciplinar diminuiu a incidência de diálise e reduziu a mortalidade por todas as causas. Ademais, após o estabelecimento de centros de teleatendimento e prevenção da DRC, houve uma drástica redução da incidência de insuficiência renal terminal em Taiwan ^(^ ^24^ ^)^ . 

 Em geral, os estudos que se propuseram a avaliar a adesão ^(^ ^17^ ^-^ ^18^ ^,^ ^20^ ^,^ ^22^ ^)^ e o autocuidado ^(^ ^16^ ^-^ ^25^ ^)^ retrataram melhora nos índices analisados, reforçando que as tecnologias de telenfermagem foram capazes de influenciar no conhecimento sobre a DRC ^(^ ^16^ ^-^ ^19^ ^,^ ^21^ ^-^ ^22^ ^,^ ^24^ ^)^ , na adesão medicamentosa ^(^ ^16^ ^-^ ^18^ ^,^ ^20^ ^-^ ^21^ ^,^ ^23^ ^-^ ^25^ ^)^ , na dieta ^(^ ^16^ ^-^ ^18^ ^,^ ^21^ ^,^ ^24^ ^-^ ^25^ ^)^ , no monitoramento da pressão arterial ^(^ ^18^ ^,^ ^23^ ^)^ , na prática de exercícios ^(^ ^16^ ^-^ ^18^ ^,^ ^21^ ^,^ ^24^ ^,^ ^25^ ^)^ e em hábitos de vida, como o ato de fumar ^(^ ^18^ ^)^ . Contudo, quando o assunto é o conhecimento da DRC, a idade elevada, a baixa escolaridade e a duração da doença influenciam o processo de aprendizagem do paciente ^(^ ^18^ ^)^ . 

 Cabe ainda destacar que as tecnologias de fácil utilização e alcance têm sua utilidade principalmente em pacientes com condições assintomáticas ^(^ ^16^ ^)^ , em que a progressão da doença pode passar despercebida devido à falta de sintomas perceptíveis, e ser negligenciada. Portanto, esse recurso transmite ao paciente informação e conhecimento necessários para o entendimento da DRC, sua progressão e gravidade, favorecendo a adesão ao tratamento e o seu protagonismo na melhoria da qualidade de vida ^(^ ^16^ ^,^ ^20^ ^)^ . Outrossim, sob a ótica profissional, ter uma ferramenta ao alcance das mãos que facilita o monitoramento do paciente possibilita uma intervenção mais assertiva e célere pela constante vigilância, servindo de apoio no surgimento de dúvidas e proporcionando acolhimento ao paciente ^(^ ^16^ ^)^ . 

 Por fim, é importante considerar o público-alvo na utilização de novas tecnologias ^(^ ^16^ ^)^ , sobretudo quando se envolve a necessidade de um conhecimento digital prévio à sua utilização, como em casos de aplicativos, *websites* ou teleconferências. 

### O papel da enfermagem perante as tecnologias de telessaúde

 A telenfermagem não se restringe à teleconsulta, podendo ser utilizada para a realização de casos clínicos, gerenciamento de pessoal, capacitação da equipe e atendimento à comunidade. É especialmente útil para enfermeiros que não estão fisicamente presentes, mas cuja orientação é fundamental para a prestação de cuidados seguros, garantindo privacidade e confidencialidade em um ambiente confortável ^(^ ^30^ ^)^ . 

 Dentre os resultados da revisão, o enfermeiro demonstrou ser uma peça central da prática de telessaúde. Em estudos nos quais a telenfermagem foi o foco ^(^ ^16^ ^-^ ^17^ ^,^ ^25^ ^)^ , seu papel enquanto líder no gerenciamento da tecnologia foi essencial para sua implementação e efetividade no cuidado ao paciente com DRC. Mesmo na equipe multiprofissional, o enfermeiro mantém a relevância do seu papel na telessaúde, fornecendo suporte, orientação, educação e monitoramento do estado de saúde ^(^ ^18^ ^,^ ^23^ ^-^ ^24^ ^)^ . 

 O uso da telenfermagem facilita a implantação de diretrizes e capacitação entre os cuidadores ^(^ ^27^ ^)^ e fornece educação e conscientização ao paciente sobre o envolvimento ativo em seu próprio cuidado, oferecendo as melhores práticas para o engajamento do seu autocuidado (autogestão) ^(^ ^22^ ^,^ ^25^ ^)^ . Esta se revela como prática de pesquisa, de cuidado e de gerenciamento, que pode promover a adesão, a melhoria do acesso ao cuidado e a segurança do paciente, assim como a criação de uma rede de comunicação e informação interprofissional, incentivando esta nova prática e a produção e inovação tecnológica ^(^ ^28^ ^)^ . 

 Para tanto, é benéfico o enfermeiro conhecer seu paciente e entender que, apesar de toda a mudança que a doença acarreta, existe uma vida além da DRC. O enfermeiro também precisa cuidar de seus próprios valores e atitudes quando se cria uma relação mútua com o indivíduo ^(^ ^29^ ^)^ . 

 Destaca-se que as implicações em conscientizar o envolvimento do paciente, apoiando práticas de cuidado que promovam seu engajamento e empoderamento, além de contribuir para a reflexão da postura do paciente renal frente à vigilância e controle de seu próprio tratamento, fomentam o cuidado em saúde, como resultado da parceria entre profissionais e usuários, de forma a colaborar para romper o status do “paciente passivo”, subjacente ao modelo de cuidado centrado na doença, tão presente nos serviços de saúde ^(^ ^31^ ^)^ . 

 Em suma, diante da potencialidade de atendimento à distância, o trabalho remoto se apresenta como uma inovação na prática da enfermagem e na área da saúde, ao mesmo tempo que um desafio aos profissionais ^(^ ^32^ ^)^ , que oportuniza uma melhor organização do serviço de saúde em uma situação de crise, como aconteceu na pandemia de covid-19, permitindo que as partes envolvidas no cuidado aos pacientes pudessem assisti-los de forma segura e com qualidade. 

 Considerando que a telenfermagem ainda é uma prática em desenvolvimento em diversos países ^(^ ^33^ ^)^ , e que a maioria dos estudos não possuíam um corte temporal longitudinal, é possível observar como lacuna do conhecimento a ausência de resultados a longo prazo da implementação da teleconsulta na regressão ou estabilização da DRC, sobretudo quando consideramos o uso de inovações tecnológicas, principalmente nos estágios iniciais da doença. 

## Conclusão

Evidenciou-se que o uso da telessaúde pela enfermagem é relevante no âmbito da saúde, tanto em termos sociais quanto econômicos, especialmente considerando a gravidade da DRC como uma condição de saúde global. Seu uso é capaz de diminuir o avanço precoce da progressão da perda da função renal, porém ainda é pouco explorado pela literatura nacional e internacional, representando uma lacuna no conhecimento a ser preenchida em futuras pesquisas. Os estudos demonstraram que sua implementação auxilia e dá suporte aos profissionais da saúde, proporcionando inferências e orientações na assistência rápida, segura e efetiva, mesmo que à distância, por meio de plataformas, teleconferências, telemensagem e portais de serviços de atendimento de tecnologia em saúde.

Agindo como meio de prevenção e promoção da saúde à distância, a telenfermagem se apresenta como estratégia capaz de promover a adesão ao tratamento e ao autocuidado em pacientes com insuficiência renal crônica em tratamento conservador. Favorece a melhoria da prática clínica e do conhecimento, e o desenvolvimento de tecnologias e inovação em saúde no campo da enfermagem, contribuindo para o avanço científico e o desenvolvimento de políticas públicas na área de doenças crônicas não-transmissíveis, podendo gerar, portanto, um impacto positivo na saúde pública.

Por fim, diante das lacunas identificadas, recomenda-se a realização de futuros estudos que se proponham a acompanhar a utilização de tecnologias em saúde para a melhoria do autocuidado e adesão ao tratamento de pacientes com DRC, desde seus estágios iniciais pré-diálise até a entrada em terapia renal substitutiva.
